# Cold sintering of ceramics: interfacial mechanisms, structure–property relationships, and multifunctional applications

**DOI:** 10.1039/d6ra05306h

**Published:** 2026-08-14

**Authors:** Kaveh Rahimi Mamaghani, Nader Parvin

**Affiliations:** a Department of Materials and Metallurgical Engineering, Amirkabir University of Technology (Tehran Polytechnic) Tehran Iran krahimi@aut.ac.ir kaveh.metal85@gmail.com

## Abstract

The cold sintering process (CSP) has emerged as a promising ceramic processing route capable of achieving densification at temperatures far below those required for conventional sintering. The process relies on the combined effects of transient liquid phases, external pressure, dissolution–precipitation reactions, and subsequent thermal activation, resulting in distinctive microstructural and interfacial characteristics. Beyond low-temperature densification, CSP offers opportunities to tailor dielectric, thermal, ionic, ferroelectric, piezoelectric, and energy-storage properties through control of grain boundaries, residual phases, and defect chemistry. Despite significant progress, important questions remain regarding interfacial transport mechanisms, transient phase evolution, grain-boundary behavior, and long-term stability. Unlike previous reviews that primarily focus on densification mechanisms or specific material systems, this article establishes an interface-centered framework linking grain-boundary chemistry, defect evolution, and coupled transport phenomena to multifunctional performance in cold-sintered ceramics. This review examines the physicochemical mechanisms governing CSP and their relationships with microstructural development and functional performance. Particular attention is given to ionic transport, thermal conduction, dielectric polarization, and defect-mediated electrical behavior. Alongside the process–interface–transport–property paradigm, emerging applications, characterization approaches, modeling efforts, and challenges associated with scalability and sustainable manufacturing are also discussed.

## Abstract 2

An interface-centered framework links dissolution–transport–precipitation and engineered grain boundaries to ionic, electrical, thermal, and mechanical transport, enabling multifunctional ceramics at low temperatures.

## Graphical abstract

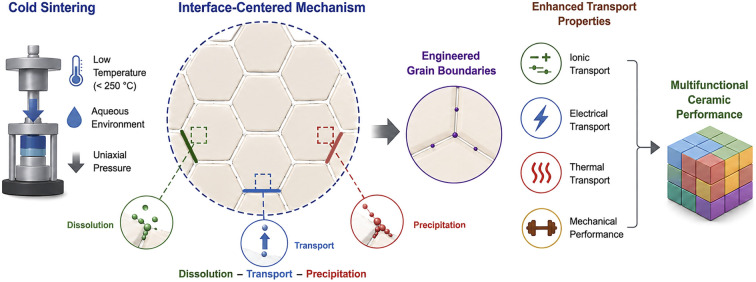

## 1. Introduction

**Fig. 1:**
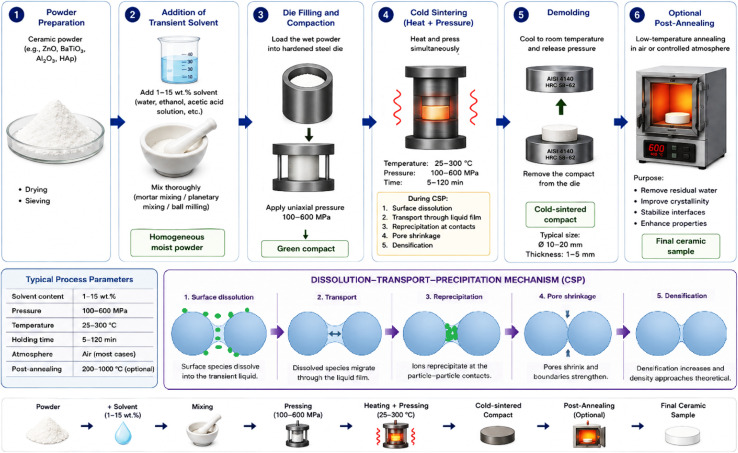
Overview of the CSP for fabricating ceramic components from powder precursors. The process includes powder preparation, transient solvent addition, die filling and compaction, simultaneous application of pressure and moderate temperature, densification through a dissolution–transport–precipitation mechanism, demolding, and optional post-annealing. The combination of solvent-mediated mass transport and pressure-assisted densification enables the production of dense ceramic materials at temperatures typically below 300 °C.

The development of advanced ceramic materials has traditionally been constrained by the high temperatures required for densification and microstructural consolidation.^1^ Conventional sintering generally involves temperatures above 1000–1600 °C, where diffusion-driven processes promote densification, grain growth, pore elimination, and phase stabilization. Although these approaches have enabled the production of high-performance structural and functional ceramics, they are associated with substantial energy consumption, volatilization of thermally sensitive constituents, excessive grain growth, residual stresses, and limited compatibility with polymers, metals, and other temperature-sensitive materials.^2–5^ For example, conventional solid-state sintering of ZnO ceramics requires 59 080 kJ g^−1^ of energy consumption, while conventional sintering of BaTiO_3_ powder demands 2800 kJ g^−1^.^6^ These limitations have become increasingly important as ceramic technologies move toward multifunctional integration, miniaturization, and sustainable manufacturing. The cold sintering process (CSP) has emerged as a significant alternative to conventional ceramic processing by enabling densification at temperatures typically below 300 °C.^7^ The fundamental processing sequence of the CSP is illustrated in Fig. 1. Unlike traditional sintering, which is dominated by solid-state diffusion, CSP combines transient liquid phases, externally applied pressure, dissolution–precipitation processes, and moderate thermal activation.^8,9^ This approach enables substantial densification at dramatically reduced temperatures and has been successfully applied to dielectric ceramics,^10^ microwave materials,^11^ solid electrolytes,^12^ piezoelectrics,^13^ thermoelectrics,^14^ ferrites,^15^ composites,^16^ and energy-storage systems.^17^ Notably, CSP can reduce the sintering temperature of metakaolin ceramics from ∼1400 °C to 240 °C while achieving 90% relative density,^18^ and densify BaTiO_3_ ceramics at an ultra-low temperature of 180 °C with relative densities exceeding 96%.^17^ The energy consumption of CSP equipment is only 0.86 kW h, which is 6.66 times lower than that of conventional thermal sintering (5.73 kW h).^19^ Beyond these established applications, CSP has recently being explored as an enabling processing platform for emerging multifunctional ceramic systems, including hard metals,^5^ quantum ceramics^20^ and sustainable, self-healing ceramic concepts.^21^ The significance of CSP extends beyond temperature reduction.^22^ The process introduces distinct pathways for mass transport, defect redistribution, grain-boundary evolution, and interfacial phase formation. Solvent-mediated transport mechanisms play a central role during densification, while subsequent thermal treatments influence crystallization, residual stress relaxation, and interfacial stabilization.^3^ As a result, cold-sintered ceramics often exhibit microstructures that differ substantially from those produced by conventional sintering, leading to unique electrical, dielectric, thermal, and ionic transport characteristics.

A major advantage of CSP is its ability to suppress volatilization and preserve metastable or compositionally sensitive phases.^23,24^ This is particularly important for alkali-containing ceramic systems, where volatilization during high-temperature processing can induce defect formation and degrade functional performance.^25^ By reducing the thermal budget and limiting defect generation, CSP offers opportunities to produce dense ceramics with refined microstructures and enhanced functional properties. For instance, cold-sintered ZnO ceramics have achieved relative densities exceeding 99% with grain sizes below 200 nm,^26^ while a two-step CSP process yielded ZnO nanocrystalline ceramics with 97.5% relative density and an average grain size of 39 nm from 30 nm powder.^27^ Recent studies have demonstrated improvements in dielectric and energy-storage performance through grain refinement, increased breakdown strength, and controlled defect chemistry achieved by CSP combined with optimized post-annealing treatments.^28–30^ Cold-sintered BaTiO_3_ ceramics exhibit high breakdown strength of 90 kV cm^−1^, energy storage density of 1.45 J cm^−3^, and energy storage efficiency of 85.6%, which achieve a dielectric constant of 2332 at 1 kHz with a mean grain size as low as 122 nm after annealing.^17^ Despite considerable progress, several fundamental aspects of CSP remain insufficiently understood. The interactions among pressure, liquid-phase chemistry, dissolution–precipitation kinetics, defect migration, and grain-boundary evolution are highly material dependent and often complex.^31,32^ Furthermore, the influence of residual intergranular phases, metastable interfaces, and heterogeneous stress distributions on long-term performance and reliability remains an active area of investigation.^33^ Transport phenomena represent a particularly important aspect of cold-sintered ceramics. Functional performance is governed by processes such as ionic migration, charge transport, dielectric polarization, phonon conduction, and interfacial relaxation.^34^ Because CSP generates interface-rich microstructures and complex grain-boundary environments, transport behavior often differs from that observed in conventionally sintered materials. Grain-boundary phases may enhance densification while limiting thermal conductivity, whereas grain refinement can improve dielectric breakdown strength but alter thermal and ionic transport pathways.^35^ Understanding these coupled effects is essential for the design of high-performance cold-sintered ceramics.

**Fig. 2:**
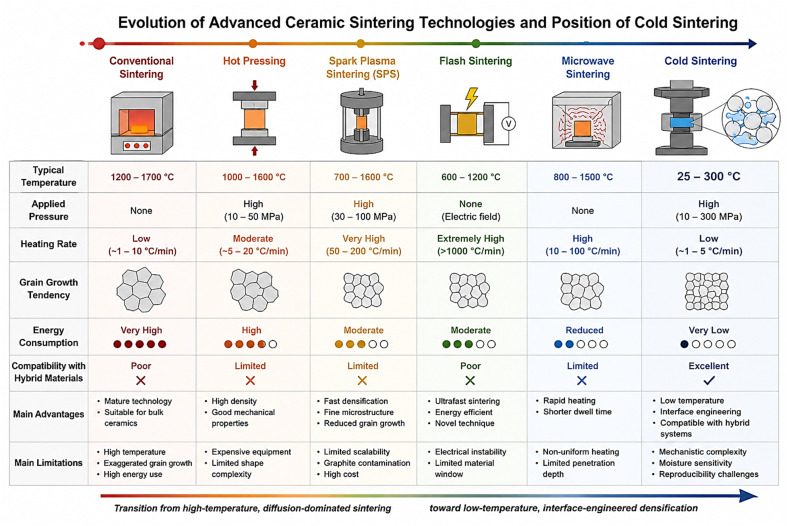
Schematic comparison of major ceramic sintering technologies including conventional sintering, spark plasma sintering (SPS), flash sintering, microwave sintering, and CSP. The figure highlights differences in processing temperature, pressure, grain-growth behavior, energy consumption, and compatibility with multifunctional hybrid systems. CSP uniquely combines low-temperature densification with interface-controlled microstructural engineering.

The low processing temperatures associated with CSP also create opportunities for integrating ceramics with polymers, metals, carbon-based materials, and other temperature-sensitive components.^9^ These capabilities are attractive for multilayer electronic devices,^20,36^ hybrid composites,^37^ embedded energy systems, and additive manufacturing technologies. In addition, the substantial reduction in processing temperature offers potential benefits in terms of energy efficiency and environmental sustainability.^38^ Life cycle assessment studies have demonstrated that CSP significantly reduces global warming potential, energy demand, and water consumption compared to traditional manufacturing approaches.^19,31^ Although several reviews have summarized the fundamentals and applications of CSP,^4,7,23,34,39–42^ the relationships between densification mechanisms, interfacial transport phenomena, and multifunctional performance remain insufficiently addressed. Unlike conventional ceramic processing, where densification is primarily controlled by thermal activation, CSP creates a materials platform in which grain-boundary chemistry, interfacial structure, and nanoscale transport pathways become dominant design variables. From a broader perspective, the rapid expansion of CSP research has produced an increasingly diverse body of literature spanning dielectric materials, electrochemical ceramics, structural ceramics, composites, and multifunctional hybrid systems. Nevertheless, much of the published work remains focused on demonstrating successful densification or individual property improvements, whereas comparatively fewer studies systematically compare the governing interfacial mechanisms responsible for these outcomes. This fragmentation has made it difficult to distinguish universal transport principles from material-specific phenomena. Therefore, rather than treating CSP simply as a low-temperature processing technology, the present review adopts an interface-centered analytical framework to critically evaluate similarities, discrepancies, and emerging design principles across different ceramic systems. Consequently, the future development of CSP may be viewed not simply as low-temperature densification but as a transition toward interface-driven ceramic engineering, where thermal, electrical, ionic, and mechanical properties are programmed through controlled interfacial architectures. Fig. 2 compares the operating principles and processing windows of major ceramic sintering technologies, while Table 1 summarizes the characteristics of the principal ceramic densification routes. This review therefore focuses on the links between densification chemistry, interfacial transport processes, microstructural evolution, and functional properties in cold-sintered ceramics, with particular emphasis on dielectric, thermal, ionic, and energy-storage applications. Also, rather than presenting interface-controlled transport merely as a descriptive concept, this review employs it as an analytical framework to systematically compare different ceramic systems, explain variations in densification behavior and functional performance, and identify the material classes that benefit most from CSP.

**Table 1:** Comparison of major advanced ceramic sintering technologies with respect to processing conditions, grain-growth behavior, energy consumption, and compatibility with multifunctional hybrid systems

Technique	Typical temperature (°C)	Pressure	Heating rate	Grain growth	Energy consumption	Hybrid compatibility	Main advantages	Main limitations	Key ref.
Conventional sintering	1200–1700	No	Low	Severe	Very high	Poor	Mature technology	High energy consumption	6 and 234
Hot pressing	1000–1600	High	Moderate	Moderate	High	Limited	High density	Expensive tooling	235 and 236
SPS	700–1600	High	Very high	Reduced	Moderate	Limited	Fast densification	Limited scalability	58, 237 and 238
Flash sintering	600–1200	Electric field	Extremely high	Reduced	Moderate	Poor	Ultrafast sintering	Thermal instability	239 and 240
Microwave sintering	800–1500	No	High	Moderate	Reduced	Limited	Rapid heating	Nonuniform heating	155 and 241
CSP	25–300	High	Low	Minimal	Very low	Excellent	Low-temperature integration	Mechanism complexity	8, 40 and 42

### 1.1. Review methodology

This review was developed through a comprehensive survey of the published literature on the CSP. Relevant publications were identified primarily using the Web of Science, Scopus, and Google Scholar databases, covering the period from the introduction of CSP in 2016 through early 2026. The literature search employed combinations of keywords including cold sintering, cold sintering process, ceramics, densification, interface, grain boundary, transport phenomena, dielectric ceramics, solid electrolytes, thermal transport, energy storage, and composites. Priority was given to peer-reviewed research articles reporting significant advances in processing mechanisms, interfacial phenomena, structure–property relationships, and functional applications of CSP. Representative review articles were also considered to provide historical context and identify major research trends. Rather than providing an exhaustive systematic review, this article aims to present a critical and integrated perspective on the current state of CSP by emphasizing the mechanistic role of interfacial transport and its influence on microstructural evolution and multifunctional performance. The selected literature was therefore evaluated based on its scientific relevance, originality, and contribution to the understanding of interface-controlled CSP. Whenever multiple studies reported similar phenomena, representative and highly cited publications were preferentially selected, while recent contributions were included to highlight emerging developments and future research directions.

## 2. Interface-centered framework for understanding cold sintering

**Fig. 3:**
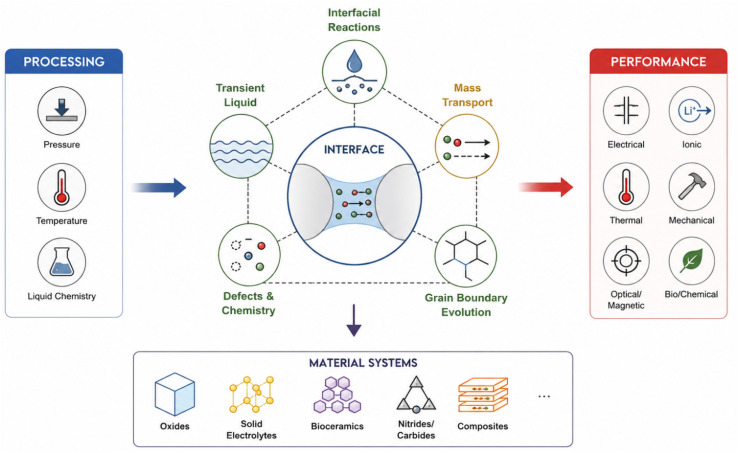
Interface-centered framework illustrating how processing variables regulate interfacial transport, which governs microstructural evolution and ultimately determines the multifunctional performance of cold-sintered ceramics. The framework provides a unified basis for interpreting and comparing different.

Although the classical processing–structure–property paradigm has long served as the foundation for understanding ceramic processing, it does not fully capture the unique characteristics of the CSP. In conventional sintering, densification is primarily governed by high-temperature bulk and grain-boundary diffusion. In contrast, CSP operates through transient liquid-assisted interfacial transport under simultaneous pressure and low-temperature conditions.^7,43^ Under these conditions, the solid–liquid interface is not merely a consequence of processing but becomes the primary region where dissolution, reprecipitation, particle rearrangement, and grain-boundary evolution occur.^44^ Consequently, interfacial phenomena directly govern microstructural development and the resulting functional properties of cold-sintered ceramics. Rather than replacing the classical processing–structure–property framework, the present review extends it by explicitly incorporating interfacial transport as the central mechanism connecting processing variables with microstructural evolution and multifunctional performance. This interface-centered perspective provides a unified framework for interpreting the diverse literature on CSP, enabling consistent comparison of different ceramic systems despite substantial differences in composition, transport species, and targeted applications. Within this framework, pressure, transient liquid chemistry, particle characteristics, and post-treatment collectively regulate interfacial reactions, which subsequently determine grain-boundary evolution, densification behavior, defect chemistry, and ultimately functional performance. Fig. 3 illustrates the proposed interface-centered framework. Unlike the traditional linear processing–structure–property relationship, the revised framework explicitly recognizes interfacial transport as the governing link between processing parameters and material performance. The framework further highlights that the dominant interfacial mechanisms differ among ceramic systems while remaining the common driving force responsible for densification and property optimization in CSP. The comparison presented in Table 2 demonstrates that the proposed framework performs an analytical rather than descriptive role. Although different ceramic systems rely on distinct transport species and target different functional properties, they exhibit a common mechanistic pathway in which processing variables regulate interfacial reactions that subsequently control densification, grain-boundary evolution, and property development. This observation explains why CSP can be successfully applied to structurally and functionally diverse ceramic materials despite substantial differences in chemistry and application requirements. Furthermore, the framework provides a practical basis for identifying the processing variables that are expected to exert the greatest influence in each material class, thereby facilitating the rational design and optimization of future cold-sintered ceramics.

**Table 2:** Interface-centered interpretation of representative cold-sintered ceramic systems. Although the dominant transport species and target properties differ among materials, interfacial transport provides a common mechanistic basis for understanding densification, microstructural evolution, and functional performance

Material system	Dominant interfacial process	Primary transport mechanism	Microstructural consequence	Main property enhancement	Key ref.
ZnO	Dissolution–reprecipitation at particle contacts	Interface-assisted ionic transport	Refined grains and high densification (>95% relative density)	Enhanced electrical conductivity and mechanical strength	58
BaTiO_3_	Grain-boundary dissolution and reprecipitation	Ionic transport *via* hydrothermal precursor solutions	Dense microstructure with fine grains (mean size ∼122 nm after annealing)	High dielectric constant (*ε*_r_ = 2332 at 1 kHz) and breakdown strength (90 kV cm^−1^)	17
LLZO	Grain-boundary densification *via* transient liquid phase	Li-ion transport	Reduced interfacial resistance; good grain-boundary interfaces	Enhanced ionic conductivity (∼10^−4^ S cm^−1^)	83
Hydroxyapatite	Interface-assisted particle rearrangement with biomineralization	Dissolution–reprecipitation	Nanostructure preservation; uniform nanosized grains	Improved bioactivity, mechanical integrity, and optical transparency (81.6% at 550 nm)	103 and 242
Composite ceramics	Interfacial phase coupling *via* transient solvent	Mixed transport (ceramic + polymer)	Homogeneous phase distribution; high-density composites	Multifunctional performance (mechanical, electric, dielectric)	56

Although interfacial transport provides a useful unifying framework for interpreting CSP across diverse ceramic systems, it should not be regarded as a universal predictive model. The relative importance of dissolution–reprecipitation, stress-assisted diffusion, capillary transport, grain-boundary migration, and defect redistribution varies substantially depending on ceramic chemistry, solvent composition, and processing conditions.^7^ Consequently, similar interfacial architectures may evolve through fundamentally different transport pathways, indicating that the proposed framework should be viewed as a mechanistic guide rather than a universally applicable processing model. A broader analysis of the available literature also reveals that the field is gradually shifting from optimizing individual processing parameters toward engineering interfaces as functional design elements. Earlier studies primarily investigated the effects of pressure, temperature, or solvent chemistry independently, whereas recent investigations increasingly focus on how these variables collectively modify grain-boundary chemistry, defect distributions, and transport pathways.^39^ This transition suggests that future advances in CSP are more likely to arise from interface-by-design strategies than from further optimization of individual processing parameters alone. However, the literature does not yet provide a consensus regarding the dominant transport mechanism governing all cold-sintered ceramics. While ZnO, hydroxyapatite, and several oxide ceramics are generally interpreted within the classical dissolution–reprecipitation framework, other materials (including glass-ceramics, composite systems, and certain solid electrolytes) exhibit evidence of stress-assisted densification, localized plastic deformation, or solvent-assisted grain-boundary diffusion.^43^ These observations indicate that the governing mechanism is inherently material dependent rather than universal. An important knowledge gap emerging from the current literature is the absence of quantitative descriptors capable of predicting the dominant interfacial transport mechanism for a given ceramic system. Existing studies largely remain empirical, with processing windows established experimentally rather than through predictive mechanistic criteria. Developing quantitative relationships linking solvent chemistry, defect thermodynamics, interfacial energies, and transport kinetics therefore represents one of the most important future research priorities for CSP. Rather than viewing interfaces simply as regions where densification occurs, the evidence increasingly supports their role as active structural entities that simultaneously regulate ionic transport, dielectric polarization, phonon scattering, defect evolution, and long-term functional stability. This conceptual shift distinguishes CSP from conventional ceramic processing and provides the fundamental rationale for the interface-centered framework proposed in this review.

## 3. Fundamental principles and densification mechanisms of cold sintering

The CSP differs fundamentally from conventional ceramic densification because mass transport is dominated by solvent-assisted interfacial processes rather than thermally activated solid-state diffusion.^8^ In conventional sintering, densification is driven by lattice diffusion, grain-boundary diffusion, vapor transport, and viscous flow at temperatures typically exceeding 0.7 *T*_m_, where *T*_m_ is the melting temperature in Kelvin.^22,44^ Although effective for achieving high density, these processes often require substantial energy input and may promote grain growth, volatilization of unstable species, and microstructural coarsening.^9^ In contrast, CSP enables densification at temperatures generally below 300 °C through the combined action of a transient liquid phase, external pressure, dissolution–precipitation reactions, particle rearrangement, and moderate thermal activation.^11^ Mass transport occurs through localized liquid-mediated pathways, allowing rapid densification under conditions where conventional diffusion mechanisms remain largely inactive.^44^ Consequently, CSP can be viewed as a hybrid processing strategy that integrates hydrothermal chemistry, pressure-assisted consolidation, and low-temperature structural stabilization. In other words, the available evidence indicates that the relative contribution of each densification mechanism is not constant across ceramic systems. While solvent-assisted mass transport dominates highly soluble oxide ceramics, its contribution decreases in chemically stable or multiphase materials, where stress-assisted particle rearrangement, localized deformation, and interfacial diffusion may become equally important. Consequently, describing CSP solely as a dissolution-controlled process oversimplifies the complexity of the underlying transport phenomena.

**Fig. 4:**
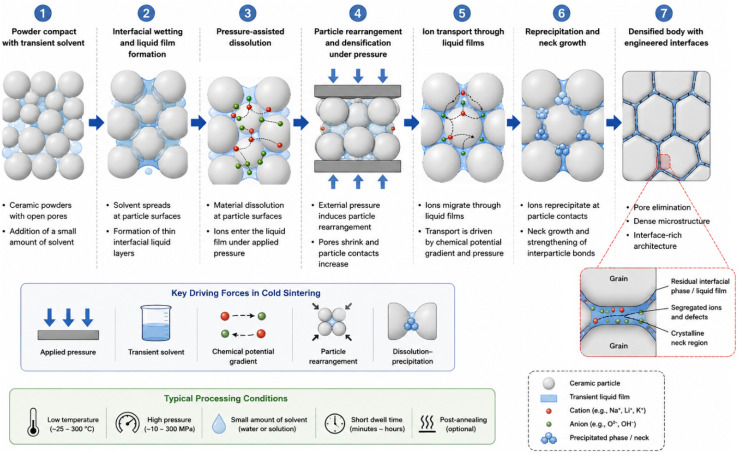
Schematic illustration of the fundamental mechanisms involved in CSP. The process includes transient solvent-assisted wetting, pressure-enhanced dissolution of particle surfaces, interfacial ion transport, particle rearrangement, reprecipitation, and low-temperature densification. The resulting ceramics typically exhibit fine-grained microstructures with interface-rich architectures and suppressed grain growth.

The process typically begins with the addition of a transient liquid phase to the ceramic powder. Water is the most widely used solvent because of its high dielectric constant, strong polarity, low cost, and environmental compatibility.^45–47^ Depending on the material system, organic,^48^ acidic,^49^ alkaline,^50^ saline,^51^ or mixed-solvent^52^ solutions may also be employed to modify dissolution kinetics and phase stability. Following liquid addition, the powder compact is subjected to uniaxial pressure and moderate heating. During this stage, capillary-assisted particle rearrangement, surface dissolution, ion transport, localized supersaturation, precipitation, and pore elimination occur simultaneously.^53^ The dissolution–precipitation mechanism is widely accepted as the primary densification pathway in many cold-sintered ceramics.^4^ Although dissolution–precipitation is generally considered the dominant densification mechanism, increasing evidence suggests that CSP cannot be described by a single universal process. Depending on the ceramic chemistry, solvent system, and processing conditions, densification may also involve pressure-assisted plastic deformation, particle fragmentation, stress-enhanced diffusion, capillary forces, and localized hydrothermal reactions.^54^ Consequently, CSP should be regarded as a spectrum of coupled mechanisms rather than a purely dissolution-driven phenomenon. Developing predictive models capable of quantifying the relative contributions of these mechanisms remains one of the central challenges in the field. Nevertheless, consensus has not yet been reached regarding the quantitative contribution of dissolution–reprecipitation relative to other transport mechanisms. For example, TEM observations provide strong evidence for dissolution-assisted neck formation in ZnO and several oxide ceramics, whereas other systems exhibit microstructural features consistent with stress-enhanced diffusion or pressure-assisted particle deformation.^44^ These observations suggest that multiple transport pathways may operate simultaneously, with their relative importance determined by ceramic chemistry, solvent composition, and local stress distribution rather than by a single universal mechanism.^55^ Under pressure and moderate temperature, highly stressed regions such as particle asperities and contact points preferentially dissolve into the transient liquid phase.^47^ The dissolved species subsequently migrate through intergranular liquid films and reprecipitate at regions of lower chemical potential, promoting neck formation and pore filling.^56^ As densification progresses, the amount of free liquid decreases while interparticle bonding increases.^40^ This sequence of dissolution, transport, and reprecipitation is schematically illustrated in Fig. 4.

The efficiency of this mechanism depends on several interrelated parameters, including solvent composition, pH, ionic concentration, particle size, surface energy, applied pressure, heating rate, and processing temperature.^40,57^ Among these factors, solvent chemistry is particularly important because it controls both the solubility of surface species and the mobility of dissolved ions.^25,44^ For instance, ZnO shows appreciable solubility under both acidic and alkaline conditions; however, the (Zn(CH_3_COO))^+^ complex promotes ZnO reprecipitation more effectively than (Zn(OH)_4_)^2−^, leading to superior CSP performance in the CH_3_COOH system.^58^ A relative density exceeding 95% is achieved at 100 MPa/150 °C in the CH_3_COOH system, whereas the NaOH system requires more stringent conditions (150 MPa/200 °C) to reach comparable densification. Minor changes in solution chemistry can significantly influence densification kinetics, precipitation behavior, and intermediate-phase formation. In alkali-containing ceramics, such as NaNbO_3_-based systems, partial dissolution of alkali species may facilitate transport while simultaneously affecting stoichiometry and defect chemistry.^59,60^ External pressure plays a central role throughout densification. It enhances particle packing, reduces pore volume, increases the number of particle contact sites, and promotes intimate interfacial contact.^61^ Pressure also increases local chemical potential gradients at stressed asperities, accelerating dissolution and subsequent reprecipitation. As a result, the applied pressure strongly influences final density, grain-boundary structure, residual porosity, and defect distributions.^44^ Recent studies on cold-sintered SrFe_12_O_19_ have shown that interfacial dissolution–precipitation processes govern densification during CSP. The acetic acid–ethanol transient solvent promotes the formation of iron-carboxylate intermediates at particle contacts, which facilitate particle bonding and densification.^62^ Collectively, these findings demonstrate that solvent chemistry functions not merely as a processing aid but as an active design parameter controlling interfacial reactions, defect evolution, and densification kinetics.^58^ However, the optimum solvent composition remains highly material dependent. Solvents that enhance dissolution in one ceramic system may induce undesirable secondary phases, compositional deviations, or pore formation in another, emphasizing the need for material-specific solvent design rather than universal processing protocols.^62–64^ However, excessive solvent concentration or elevated temperatures lead to Fe_2_O_3_ formation and pore generation, highlighting the critical role of transient grain-boundary chemistry in controlling microstructural evolution and functional properties. From a thermodynamic perspective, CSP may be interpreted as a process that exploits local reductions in activation barriers through transient liquid-mediated transport. The liquid phase effectively provides a high-mobility pathway that circumvents the long-range lattice diffusion required during conventional sintering, thereby enabling significant densification under conditions far below those predicted by classical solid-state sintering theory.

Despite the low processing temperature, CSP alone does not always produce fully stabilized ceramic bodies. Post-annealing is frequently employed to remove residual liquid-derived species, improve crystallinity, strengthen interparticle necks, and complete structural stabilization.^65–67^ Therefore, CSP is often best regarded as a two-stage process consisting of solvent-assisted densification followed by low-temperature thermal stabilization. The necessity of post-annealing also highlights an important distinction between laboratory-scale densification and practical materials engineering. While CSP successfully minimizes the thermal budget required for consolidation, many functional properties (including electrical conductivity,^65^ dielectric performance,^68,69^ and mechanical integrity^70^) remain strongly dependent on subsequent thermal stabilization. Consequently, reported property enhancements should be interpreted in the context of the complete processing route rather than CSP alone.^71^ The balance between these stages significantly affects the final degree of consolidation. Importantly, these transformations occur at substantially lower thermal budgets than those required for conventional sintering, preserving the low-temperature processing advantages of CSP. Densification mechanisms become more complex in multiphase ceramics and composite systems (Table 3). Differences in solubility, surface chemistry, mechanical response, and phase stability among constituent materials can generate heterogeneous dissolution and precipitation behavior during processing.^32^ In some systems, one phase may facilitate densification of another through preferential dissolution or enhanced mass transport, whereas interfacial incompatibilities may lead to incomplete consolidation and residual porosity.^34^ These effects highlight the importance of understanding material-specific densification pathways when extending CSP to complex ceramic architectures.

**Table 3:** Proposed densification and transport mechanisms involved in CSP and their associated driving forces and limitations^39,44,201,220^

Mechanism	Description	Driving force	Supporting evidence	Key limitation	Key ref.
Dissolution–precipitation	Solvent-mediated dissolution and reprecipitation	Chemical potential gradient	TEM/interfacial studies	Material-dependent solubility	39 and 44
Plastic deformation	Local particle deformation	Applied pressure	Densification behavior	Limited for brittle systems	201 and 243
Capillary transport	Liquid-assisted particle rearrangement	Surface energy minimization	Wetting observations	Solvent sensitivity	32 and 220
Interface-assisted diffusion	Accelerated grain-boundary transport	Interfacial energy	Fine-grain evolution	Difficult direct observation	34 and 244
Stress-enhanced transport	Pressure-driven mass redistribution	Mechanical stress	High-density formation	Multiphysics complexity	44 and 55

**Fig. 5:**
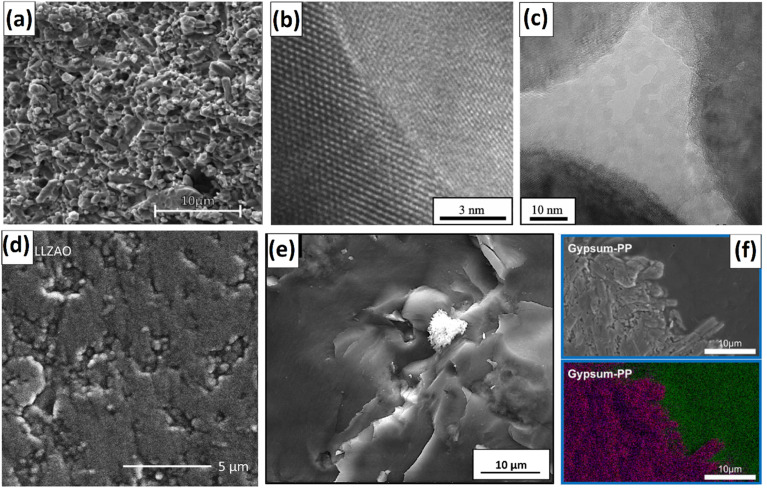
Representative experimental microstructures illustrating the characteristic structural features and densification mechanisms produced by the CSP across different ceramic systems. (a) Fracture SEM micrograph of cold-sintered ZnO demonstrating dense particle packing with suppressed grain growth compared with conventional sintering.^73^ (b) High-resolution TEM image showing a clean recrystallized ZnO grain boundary without detectable amorphous intergranular phases after CSP.^74^ (c) High-resolution TEM image of a ZnO triple junction revealing dissolution–reprecipitation-assisted pore filling and the formation of newly recrystallized material during densification.^74^ (d) Backscattered SEM image of cold-sintered Li-rich garnet (LLZAO) exhibiting a dense phase-pure microstructure with fine grains and no observable secondary phases.^75^ (e) SEM micrograph of cold-sintered hydroxyapatite/niobium-phosphate glass (HAp/BG) ceramics showing enhanced consolidation, reduced porosity, and homogeneous particle bonding.^76^ (f) SEM micrograph and corresponding EDX false-color elemental map of a cold-sintered gypsum/polypropylene composite illustrating the formation of a co-continuous ceramic/polymer microstructure with interconnected phases, where calcium (purple) identifies the ceramic framework and carbon (green) identifies the polymer phase.^37^ Panels (a–f) are reproduced or adapted from the cited references under their respective Creative Commons licenses.

Although the dissolution–reprecipitation mechanism provides the fundamental framework for understanding CSP, direct experimental observations are essential to verify the resulting microstructural evolution. Representative SEM and TEM micrographs from different classes of cold-sintered ceramics are presented in Fig. 5 to illustrate the characteristic structural features produced during densification. Despite the diversity of ceramic systems, these experimental observations consistently reveal suppressed grain growth, clean recrystallized grain boundaries, dissolution–reprecipitation-assisted pore filling at triple junctions, dense phase-pure microstructures, homogeneous particle bonding, and the formation of co-continuous ceramic/polymer architectures. Together, these representative microstructures demonstrate the broad applicability of the CSP mechanism across structural, electroceramic, biomedical, and composite materials.^72^Fig. 5 provides direct experimental evidence supporting the densification mechanisms discussed above. The ZnO micrographs demonstrate that significant densification can be achieved while suppressing abnormal grain growth,^73^ whereas high-resolution TEM observations reveal clean recrystallized grain boundaries and dissolution–reprecipitation-assisted pore filling at triple junctions.^74^ Similar microstructural characteristics are observed in LLZAO electrolytes^75^ and hydroxyapatite-based bioceramics,^76^ confirming that low-temperature processing can simultaneously preserve phase stability and promote effective particle bonding. Furthermore, the formation of co-continuous ceramic/polymer microstructures illustrates the capability of CSP to integrate dissimilar material classes into multifunctional composite architectures while maintaining interconnected phases.^37^ These representative examples collectively highlight the versatility of CSP as a universal low-temperature densification strategy. Despite the diversity of the ceramic systems presented in Fig. 5, a consistent microstructural trend emerges across the literature. CSP generally suppresses abnormal grain growth while promoting dense interface-rich microstructures through localized dissolution and reprecipitation.^77,78^ However, the resulting grain-boundary structures differ considerably among oxides, electrolytes, bioceramics, and composites, indicating that similar densification levels may arise from different interfacial transport pathways.^40^ This distinction further supports the concept that CSP is governed by material-specific interface engineering rather than by a single universal densification mechanism.

Although significant progress has been made in understanding CSP mechanisms, several questions remain unresolved. Quantitative relationships among solvent chemistry, dissolution kinetics, pressure-assisted transport, and densification efficiency are not yet fully established for many ceramic systems. Meanwhile increasing pressure generally improves particle packing and accelerates dissolution at stressed contacts, the literature also indicates diminishing returns beyond material-specific thresholds. Excessively high pressures may restrict liquid redistribution, limit transport pathways, or even introduce localized residual stresses after densification.^79^ Therefore, pressure should be optimized in conjunction with solvent chemistry and particle characteristics rather than treated as an independently beneficial variable. In addition, the evolution of transient liquid phases and their role during the final stages of pore elimination require further investigation. Addressing these challenges will require combined experimental, theoretical, and *in situ* studies capable of capturing the dynamic processes that occur during densification. In simple words, the accumulated literature indicates that CSP should be interpreted as a family of coupled transport processes rather than a single densification mechanism. Dissolution–reprecipitation provides the dominant conceptual framework for many oxide ceramics; however, growing evidence demonstrates that capillary transport, stress-assisted diffusion, localized deformation, defect redistribution, and hydrothermal recrystallization may each contribute depending on the material system and processing conditions. One of the principal challenges for future research is therefore the development of predictive mechanistic models capable of quantitatively identifying the dominant transport pathway under specific processing conditions instead of relying primarily on empirical optimization. Overall, CSP should be regarded primarily as a solvent-assisted densification strategy in which pressure, transient liquid phases, and dissolution–precipitation reactions collectively enable ceramic consolidation at exceptionally low temperatures. Understanding these densification mechanisms provides the foundation for the interfacial phenomena, microstructural evolution, and application-specific performance discussed in the following sections.

## 4. Interfacial chemistry, defect evolution, and transport phenomena in cold-sintered ceramics

**Fig. 6:**
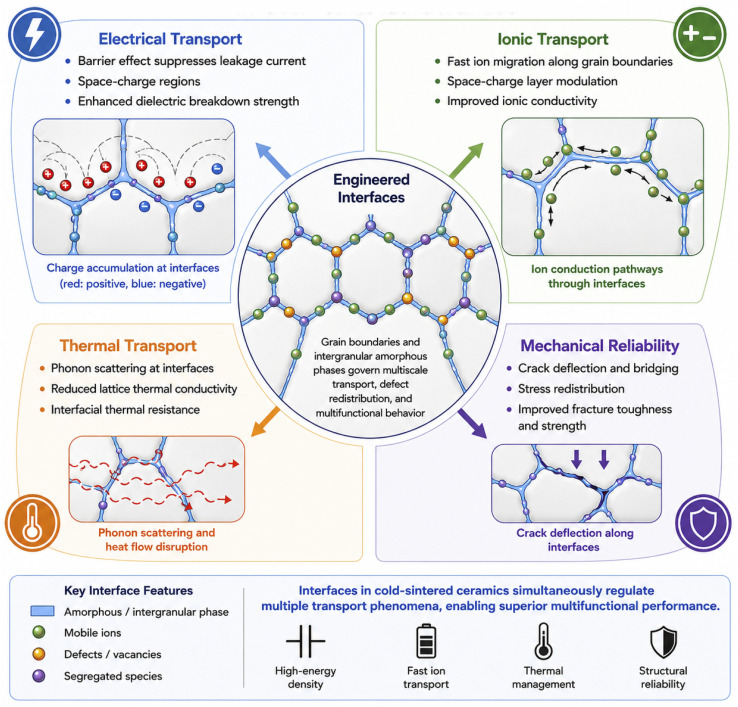
Schematic representation of coupled transport phenomena governed by engineered interfaces in cold-sintered ceramics. Grain boundaries and intergranular regions strongly influence electrical transport, ionic conduction, phonon scattering, dielectric breakdown behavior, and mechanical reliability, demonstrating the interface-dominated nature of cold-sintered systems.

The functional behavior of ceramic materials is strongly influenced by transport processes occurring across grains, grain boundaries, interfaces, and defects.^44^ In cold-sintered ceramics, these processes are particularly important because low-temperature densification generates interfacial structures that differ significantly from those produced by conventional high-temperature sintering.^56^ As a result, transport behavior is highly sensitive to grain-boundary chemistry, residual intergranular phases, defect distributions, and local structural heterogeneities.^80^ One of the defining characteristics of CSP-derived ceramics is the formation of chemically and structurally complex grain boundaries. During densification, dissolution–precipitation reactions occur within transient liquid films, generating interfacial regions that may contain segregated ions, residual solvent species, amorphous phases, or nanoscale secondary phases.^25^ These features can substantially influence transport pathways and often dominate the macroscopic response of polycrystalline ceramics. In cold-sintered BaTiO_3_ ceramics, residual amorphous phases at grain boundaries have been shown to reduce the total conductivity by up to two orders of magnitude compared to grain interiors. *In situ* synchrotron studies of ZnO cold sintering have revealed the evolution of secondary phases, including a zinc soap-type structure with a large (≈21 Å) lattice parameter retained as an intergranular component.^78^ Grain-boundary chemistry plays a central role in transport phenomena because interfacial regions frequently act either as barriers or preferential pathways for charge and mass transport.^81^ In conventionally sintered ceramics, grain boundaries are typically established through high-temperature diffusion and segregation processes. In contrast, CSP generates grain boundaries under nonequilibrium conditions, producing distinct chemical environments that may alter transport characteristics.^82^ The evolution of these interfacial structures is schematically illustrated in Fig. 6. Grain boundaries in CSP-derived ceramics should not merely be viewed as passive interfaces but as functional regions whose chemistry and structure can be deliberately engineered.^34^ Variations in residual liquid-derived species, segregated dopants, hydroxyl concentration, and local defect populations may substantially modify electrical, ionic, and thermal transport. For instance, the ionic conductivity of cold-sintered Li_7_La_3_Zr_2_O_12_ (LLZO) solid electrolytes has been shown to vary by up to three orders of magnitude depending on the concentration of LiOH residual phases at grain boundaries.^83^ Cold-sintered LLZO-based composite electrolytes using DMF as a transient liquid phase at 120 °C achieved a high conductivity of 10^−4^ S cm^−1^, while composite pellets without DMF exhibited conductivities close to 10^−9^ S cm^−1^ at ambient temperature. Consequently, grain-boundary engineering represents a promising strategy for tailoring multifunctional performance through controlled manipulation of interfacial chemistry. Although grain-boundary engineering has emerged as one of the central concepts in CSP research, the available literature indicates that the relationship between grain-boundary chemistry and functional performance is far from universal.^84^ In some oxide ceramics, chemically clean recrystallized grain boundaries facilitate electrical or ionic transport, whereas in other systems, residual intergranular phases or segregated species may either enhance transport by creating fast-conduction pathways or degrade performance by increasing interfacial resistance.^85–87^ These contrasting observations demonstrate that grain-boundary chemistry should be optimized according to the target functionality rather than minimized indiscriminately.

Defect chemistry represents another critical factor governing transport processes in oxide ceramics.^88^ Functional behavior is often highly sensitive to oxygen vacancies, cation disorder, defect complexes, and local lattice distortions. Conventional high-temperature processing frequently promotes volatilization of alkali and volatile species, resulting in increased concentrations of defect states. CSP modifies these defect-generation pathways by suppressing volatilization and limiting thermally activated defect formation.^41^ Consequently, lower concentrations of certain equilibrium defects may often be achieved compared with conventionally sintered counterparts. However, reduced thermal exposure does not eliminate defect-related phenomena. Instead, CSP introduces alternative nonequilibrium defect configurations associated with transient liquid phases,^89^ interfacial segregation, and incomplete defect equilibration.^90^ Residual hydroxyl species, dissolved ions, and solvent-derived intergranular phases may remain after densification and influence charge transport, dielectric relaxation, and interfacial polarization.^78^ The stability of these defect structures remains an important subject of ongoing investigation. An important distinction between CSP and conventional sintering therefore lies not in the elimination of defects but in the nature of the defect landscape that develops during processing.^91^ Whereas high-temperature sintering predominantly generates equilibrium defect populations through thermal activation, CSP produces metastable defect configurations governed by transient liquid chemistry, pressure-assisted transport, and incomplete equilibration.^92,93^ This difference suggests that controlling defect evolution may become equally important as achieving high densification when designing next-generation functional ceramics.

Electrical and ionic transport in cold-sintered ceramics are strongly affected by interfacial heterogeneity.^94^ Grain boundaries frequently exhibit transport characteristics that differ substantially from those of grain interiors because of compositional segregation, defect accumulation, or residual intergranular phases. As a result, grain-boundary contributions often dominate the overall transport response of cold-sintered ceramics. The balance between grain-interior and grain-boundary transport depends strongly on interfacial chemistry and defect distributions.^32^ Interfacial polarization is another important consequence of heterogeneous microstructures. Differences in conductivity and permittivity between grains, grain boundaries, and secondary phases may generate space-charge accumulation and Maxwell–Wagner-type polarization under alternating electric fields.^95^ These phenomena contribute to dielectric relaxation and frequency-dependent transport behavior. Collectively, the available evidence suggests that transport behavior in CSP-derived ceramics is increasingly governed by interfaces rather than by grain interiors alone.^85,96^ However, the relative contributions of grain-boundary conduction, space-charge effects, and residual intergranular phases remain highly material dependent. For example, solid electrolytes often benefit from enhanced interfacial ionic transport after optimized post-annealing,^26,97^ whereas dielectric ceramics frequently exhibit improved performance primarily through reduced porosity and enhanced grain-boundary insulation.^41,84^ These observations emphasize that identical microstructural features do not necessarily produce equivalent transport responses across different classes of ceramic materials. Their magnitude depends strongly on interfacial uniformity, defect concentration, and the distribution of secondary phases. The formation of space-charge regions at chemically heterogeneous interfaces may further influence transport behavior.^98^ Differences in defect concentration and electrochemical potential between grain interiors and grain boundaries can generate local charge redistribution, thereby modifying ionic mobility, dielectric response, and electrical conductivity.^99^ Understanding the evolution of these space-charge effects under low-temperature processing conditions remains an important frontier in CSP research. Despite the growing recognition of space-charge phenomena in CSP, direct experimental verification remains limited. Most interpretations are currently inferred from impedance spectroscopy, dielectric spectroscopy, or atomistic modeling rather than direct observation of local electrochemical potential gradients. Consequently, quantitative characterization of space-charge regions represents one of the major knowledge gaps limiting predictive transport models for cold-sintered ceramics. Residual stresses generated during densification may further influence transport processes. Because CSP combines pressure-assisted consolidation, liquid-phase transport, and moderate thermal activation, localized stress fields can develop within grains and at interfaces.^40,81^ These stresses may affect defect mobility, diffusion pathways, and transport characteristics, contributing additional complexity to the behavior of cold-sintered ceramics.^24^ The influence of residual stress on transport properties has also received comparatively little attention compared with densification behavior. Nevertheless, localized stress fields may modify defect mobility, alter grain-boundary structures, and influence electrochemical transport, particularly in multilayer devices and heterogeneous composites. Future investigations combining stress mapping with *operando* transport measurements may therefore provide valuable insights into stress–transport coupling in CSP-derived materials.^100^ A critical unresolved question concerns whether the nonequilibrium grain boundaries generated during CSP evolve toward equilibrium configurations during long-term operation. Such interfacial aging phenomena may significantly influence transport stability and device reliability but remain largely unexplored.

In spite of substantial progress, several challenges remain unresolved. The long-term stability of grain-boundary structures, residual intergranular phases, and nonequilibrium defect configurations under electrical, thermal, and mechanical loading is not yet fully understood. In addition, quantitative relationships linking interfacial chemistry and defect distributions to transport behavior remain insufficiently established for many ceramic systems. For cold-sintered ZnBiMnNbO varistor ceramics under 250 °C/250 MPa, the ionization activation energy of oxygen vacancies (EB = 0.32 eV) exceeded that of conventionally sintered samples (EB = 0.28 eV), where higher EB suppressed thermal excitation of carriers.^92^ Addressing these issues will require integrated experimental and computational approaches capable of resolving transport processes across multiple length and time scales. In general, CSP produces chemically and structurally distinct interfaces compared with conventional sintering. These interfaces, together with defect populations generated during low-temperature densification, govern many of the transport processes that ultimately determine material behavior.^23^ Overall, the current literature consistently demonstrates that interfacial chemistry constitutes one of the principal factors governing the functional behavior of cold-sintered ceramics. Nevertheless, the mechanisms by which chemically complex grain boundaries influence electrical, ionic, thermal, and dielectric transport remain only partially understood. The available evidence indicates that identical processing strategies may generate fundamentally different transport responses depending on defect chemistry, grain-boundary structure, and residual intergranular phases. Consequently, future progress is expected to rely less on further reducing processing temperature and more on quantitatively engineering interfacial chemistry and defect populations to tailor transport pathways for specific applications. A major challenge for future CSP research will be establishing quantitative process–interface–transport relationships capable of predicting functional performance directly from interfacial chemistry and defect evolution. Achieving this objective will require closer integration of advanced characterization techniques, multiscale computational modeling, and data-driven materials design to move beyond empirical optimization toward predictive interface engineering. Understanding their formation and evolution provides the foundation for the microstructural engineering and property optimization discussed in the following sections.

## 5. Microstructural engineering and structure–property relationships in cold-sintered ceramics

**Fig. 7:**
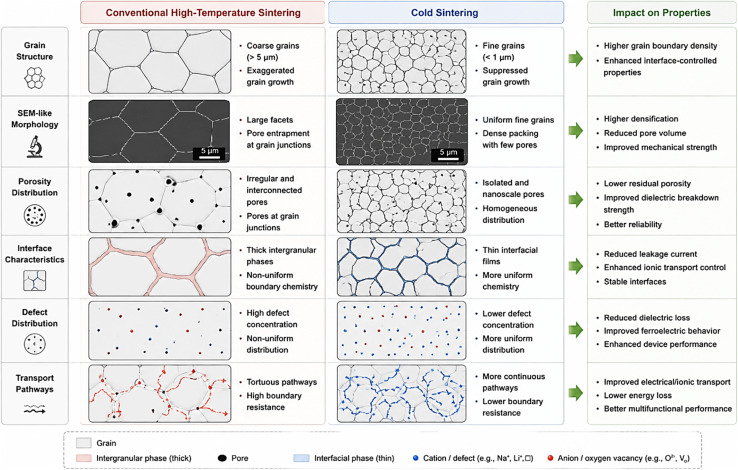
Comparison between microstructures produced through conventional high-temperature sintering and CSP. The CSP typically suppresses exaggerated grain growth and generates fine-grained heterogeneous interfaces microstructures that strongly influence transport behavior and multifunctional performance.

Microstructural engineering is one of the most effective approaches for tailoring the performance of ceramic materials because functional properties are strongly influenced by grain size, porosity, phase distribution, interfacial architecture, crystallographic organization, and microstructural heterogeneity.^1^ In cold-sintered ceramics, microstructural evolution differs significantly from that of conventionally processed materials because densification occurs through pressure-assisted, liquid-mediated transport under limited thermal exposure.^97^ Consequently, CSP enables the development of fine-grained, highly dense, and interface-rich microstructures that are often difficult to achieve using traditional high-temperature sintering routes.^56^ Representative differences between conventional high-temperature sintering and cold-sintered microstructures are illustrated in Fig. 7. Although grain refinement is widely recognized as one of the principal advantages of CSP, the available literature indicates that fine-grained microstructures do not universally translate into superior functional performance.^101^ Instead, the optimum microstructure depends strongly on the target application, as different transport phenomena respond differently to grain-boundary density, defect distribution, and interfacial chemistry. Consequently, microstructural optimization should be viewed as an application-specific design strategy rather than a universal objective. One of the most distinctive microstructural characteristics of CSP is the suppression of excessive grain growth. In conventional ceramic processing, prolonged exposure to elevated temperatures promotes grain coarsening through grain-boundary migration and diffusion-driven growth mechanisms. CSP significantly restricts these processes, allowing dense ceramics to be produced while retaining submicrometer or nanometer-scale grain structures.^40,102^ As a result, grain size becomes an important parameter governing functional behavior.

Grain size strongly influences the response of many ceramic systems. Fine-grained microstructures generally promote more homogeneous field distributions, reduce the influence of localized defects, and improve structural uniformity.^103^ In contrast, coarse-grained materials may exhibit increased heterogeneity and greater sensitivity to local microstructural fluctuations. Consequently, controlling grain size is often essential for optimizing overall performance and reliability. However, conflicting trends have been reported regarding the influence of grain refinement on functional performance. While reduced grain size generally improves dielectric breakdown strength, structural homogeneity, and mechanical reliability, excessive grain-boundary density may simultaneously increase grain-boundary resistance, suppress long-range ionic transport, or alter polarization behavior.^17,101,104,105^ These observations indicate that the optimum grain size is inherently property dependent rather than universally minimized. Porosity represents another critical microstructural parameter. Residual pores influence mechanical integrity, electrical behavior, and thermal stability by introducing local heterogeneities within the ceramic matrix.^76^ During CSP, pore elimination occurs through dissolution–precipitation processes assisted by pressure and transient liquid phases. The final porosity level depends on particle packing, solvent distribution, processing conditions, and subsequent thermal treatment.^61^ Although high relative densities can be achieved in many systems, residual nanoscale porosity may remain in materials exhibiting limited solubility or sluggish densification kinetics. Although fine-grained microstructures frequently improve dielectric breakdown strength and structural homogeneity, excessive grain-boundary area may simultaneously increase interfacial resistance and reduce transport efficiency.^14^ Consequently, microstructural optimization often involves balancing beneficial grain refinement against the potential adverse effects of increased grain-boundary density. Identifying these trade-offs represents a key objective of structure–property engineering in CSP-derived ceramics.

The distribution and morphology of pores are often as important as their overall volume fraction. Uniformly distributed nanoscale pores may have relatively minor effects on performance, whereas large or interconnected pores can act as preferential sites for stress concentration and failure initiation.^106^ Therefore, porosity optimization remains a central objective of microstructural design in CSP materials. Collectively, the literature demonstrates that porosity should be evaluated not only in terms of its volume fraction but also with respect to its morphology, spatial distribution, and connectivity.^107^ Closed nanoscale pores may exert only a limited influence on certain functional properties, whereas interconnected pore networks can severely compromise mechanical integrity, electrical insulation, and long-term reliability. Therefore, optimizing pore architecture may be as important as maximizing relative density. Phase stability and phase distribution also play important roles in determining structure–property relationships. Because CSP minimizes thermal exposure, compositionally sensitive and metastable phases can often be retained more effectively than during conventional sintering.^20,108^ Nevertheless, preserving metastable phases does not necessarily guarantee improved performance. In several material systems, retained metastable phases enhance functional properties by preventing undesirable phase transformations, whereas in others they may reduce long-term thermal stability or gradually transform during subsequent service or post-annealing.^54,109^ Consequently, phase preservation should be assessed together with its influence on operational stability rather than solely as a processing advantage. This capability is particularly valuable for complex oxide systems that may undergo decomposition, volatilization, or undesirable phase transformations at elevated temperatures. The preservation of these phases expands the accessible microstructural design space and enables new opportunities for property optimization.

Multiphase architectures further broaden the range of achievable functionalities. The distribution, connectivity, and spatial arrangement of secondary phases can significantly influence overall material behavior.^110^ Carefully designed phase architectures may generate synergistic effects that cannot be achieved in single-phase ceramics. As a result, phase-boundary engineering has become an increasingly important aspect of CSP-based microstructural design. Microstructural heterogeneity across multiple length scales is another defining feature of many cold-sintered systems.^111^ Variations in local composition, strain state, phase distribution, and interfacial architecture can generate hierarchical structures extending from the nanoscale to the microscale.^112^ Such heterogeneity frequently plays a decisive role in determining material performance and stability. Because CSP limits extensive thermal homogenization, these structural features can often be preserved and deliberately manipulated. Mechanical reliability is likewise strongly dependent on microstructure. Dense and homogeneous microstructures generally exhibit improved strength and reliability because they contain fewer critical flaws and more uniform stress distributions.^37,70^ Conversely, residual porosity, weak interfaces, and heterogeneous phase distributions may reduce structural integrity and increase susceptibility to failure.^113^ For example, cold-sintered ZnO with >95% density approximately exhibits flexural strength values in the range of 50–80 MPa,^114,115^ and fracture toughness (*K*_Ic_ = 0.57 ± 0.05 MPa m^1/2^) is ∼50% of the toughness of conventionally sintered ZnO.^101^ A broader comparison across the literature further suggests that achieving high density alone is insufficient to ensure mechanical reliability.^89^ Residual stresses, heterogeneous grain-boundary chemistry, and local variations in phase distribution may critically influence crack initiation and propagation even in highly densified ceramics.^33,116^ Future microstructural design should therefore prioritize structural uniformity and interface quality in addition to conventional densification metrics. In multilayer and composite architectures, microstructural compatibility among constituent phases becomes particularly important for long-term reliability.^117^ Future microstructural design strategies may increasingly employ inverse-design methodologies in which target functional properties are first specified and the corresponding grain-size distribution, porosity architecture, and phase arrangement are subsequently predicted using computational optimization and machine-learning approaches.

An important emerging direction is hierarchical microstructural engineering. Rather than optimizing a single parameter such as grain size, current approaches increasingly seek simultaneous control of nanoscale interfaces, mesoscale phase distributions, porosity gradients, and macroscopic architecture.^37,118^ Such strategies offer opportunities to balance multiple property requirements within a single material system and represent a promising pathway for future CSP development. Overall, microstructural engineering in cold-sintered ceramics extends beyond achieving high density and encompasses the deliberate control of grain size, porosity, phase stability, phase distribution, and hierarchical structural organization. Through these mechanisms, CSP provides a versatile platform for establishing processing–microstructure–property relationships and for designing advanced ceramic materials with application-specific performance characteristics.

**Fig. 8:**
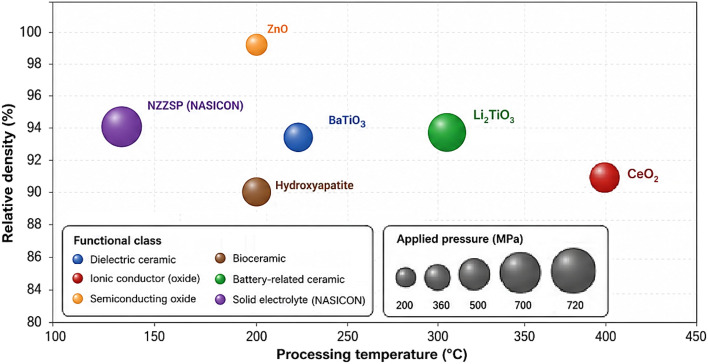
Processing temperature *versus* relative density of representative cold-sintered ceramics. Relative density is plotted as a function of cold-sintering processing temperature for experimentally verified aqueous-based CSP systems reported in the literature. Marker size is proportional to the applied uniaxial pressure during CSP, while marker color denotes the functional material class. All density values correspond to as-cold-sintered specimens directly reported in the cited publications, including BaTiO_3_,^119^ CeO_2_,^121^ ZnO,^120^ hydroxyapatite,^53^ Li_2_TiO_3_,^47^ and NZZSP/NASICON solid electrolyte.^122^

To provide a reliable quantitative comparison across different ceramic families, Fig. 8 summarizes experimentally verified cold-sintered systems for which processing temperature, applied pressure, and as-cold-sintered relative density are explicitly reported in the original literature. The selected dataset includes dielectric BaTiO_3_,^119^ semiconducting ZnO,^120^ oxygen-ion conducting CeO_2_,^121^ hydroxyapatite bioceramics,^53^ NASICON-type NZZSP solid electrolytes,^122^ and Li_2_TiO_3_ nanoceramics.^47^ These data demonstrate that CSP can achieve 90–99% relative density at temperatures between 150 and 400 °C, with the highest densification obtained for ZnO (99.43%) under a two-step 200 MPa process,^120^ while NZZSP achieved 94.3% density at only 150 °C under 720 MPa.^122^ The comparison highlights the broad applicability of CSP across dielectric, ionic-conducting, biomedical, and battery-related ceramic systems and supports the process–interface–microstructure relationships discussed throughout this review. In summary, the accumulated evidence indicates that the relationship between microstructure and functional performance in cold-sintered ceramics is considerably more complex than the traditional grain size-density paradigm commonly used in conventional ceramic processing. Instead, CSP-derived properties emerge from the combined effects of grain-boundary chemistry, hierarchical structural organization, defect populations, porosity architecture, and phase distribution. Importantly, the literature reveals no universal microstructural configuration capable of simultaneously maximizing electrical, ionic, thermal, and mechanical performance. Future advances will therefore depend on application-specific microstructural design strategies supported by quantitative process–microstructure–property models rather than empirical optimization alone. The next stage of CSP research is expected to shift from controlling individual microstructural parameters toward designing integrated microstructural architectures in which grain boundaries, defects, secondary phases, and hierarchical interfaces are simultaneously engineered to achieve multiple functional objectives. Such an approach will likely require the integration of advanced characterization, computational optimization, and machine-learning-assisted inverse materials design.

## 6. Multifunctional applications of cold-sintered ceramics

Cold sintering has enabled the fabrication of a broad spectrum of functional ceramic systems. Owing to its ability to preserve metastable phases, minimize thermal degradation, and tailor interfacial structures, CSP has been successfully applied across diverse application fields. The following subsections summarize the major application areas and highlight the underlying structure–property relationships governing their performance. An important trend emerging from recent studies is that the technological impact of CSP is increasingly determined by application-specific functional requirements rather than by densification performance alone.^77,122^ While high density remains a prerequisite for many ceramic systems, application-specific metrics, including dielectric breakdown strength, ionic conductivity, thermal conductivity, and long-term operational stability, ultimately determine the practical competitiveness of CSP-derived materials. Consequently, evaluating CSP solely in terms of densification efficiency provides an incomplete assessment of its technological potential.

### 6.1. Dielectric, ferroelectric, and energy-storage applications of cold-sintered ceramics

The growing demand for high-power and high-efficiency energy-storage technologies has stimulated extensive research into advanced dielectric ceramics capable of delivering high recoverable energy density, rapid charge–discharge rates, excellent thermal stability, and long-term operational reliability.^123^ Compared with electrochemical energy-storage systems, dielectric capacitors provide exceptionally high power density and ultrafast response times, making them attractive for pulsed-power electronics, electric transportation, aerospace systems, renewable-energy technologies, and advanced power-conditioning applications.^124^ Consequently, the development of dielectric ceramics with improved energy-storage performance has become a major research focus within the CSP community.

The energy-storage behavior of dielectric ceramics is primarily determined by their polarization–electric field (*P*–*E*) characteristics.^125^ The recoverable energy density (*W*_rec_) corresponds to the electrical energy released during the discharge process and is determined from the area enclosed between the charging and discharging branches of the *P*–*E* loop. It can be calculated as follows.^126^1
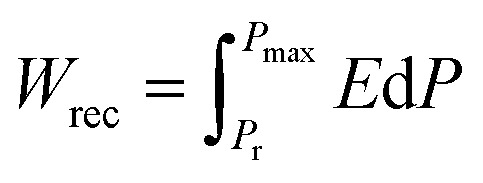
where *P*_max_ is the maximum polarization, *P*_r_ is the remanent polarization, and *E* is the applied electric field. A higher *P*_max_, lower *P*_r_, and increased breakdown strength generally contribute to a larger recoverable energy density, making these parameters key indicators for evaluating dielectric ceramics processed by CSP. So, high recoverable energy density requires the simultaneous achievement of large maximum polarization and low remanent polarization, thereby maximizing reversible energy storage while minimizing hysteresis losses.^127^ Relaxor ferroelectrics are particularly attractive in this regard because their diffuse polarization behavior promotes reversible switching and high energy-storage efficiency.^128,129^ As a result, relaxor-based systems have become one of the most actively investigated classes of CSP materials. Among the most extensively studied materials are NaNbO_3_-based relaxor ceramics.^29,30,67^ Sodium niobate exhibits attractive polarization characteristics and has emerged as a promising lead-free alternative for dielectric energy-storage applications. Recent studies have shown that compositional modification using Bi-containing complex oxides can enhance relaxor behavior, improve polarization reversibility, and increase energy-storage efficiency.^130^ These developments have established NaNbO_3_-based systems as representative examples of high-performance cold-sintered dielectric ceramics. Although remarkable improvements in recoverable energy density have been achieved, it remains important to benchmark CSP-derived ceramics against state-of-the-art conventionally sintered and thin-film dielectric systems. Such comparisons are necessary to determine whether the advantages of low-temperature processing translate into meaningful performance gains beyond manufacturing and sustainability benefits. Although enhanced breakdown strength is one of the most frequently reported advantages of CSP-derived dielectrics, the governing mechanisms remain under active debate.^131^ Current evidence suggests that grain refinement, reduced defect concentrations, and improved compositional homogeneity contribute simultaneously, but their relative importance varies across different dielectric systems.^92,132^ Establishing quantitative correlations between microstructure and breakdown behavior therefore remains an important research priority.

**Fig. 9:**
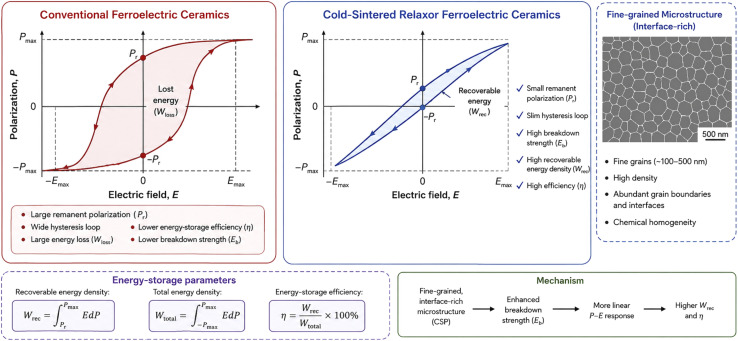
Comparison of polarization behavior and energy-storage mechanisms between conventional ferroelectric ceramics and cold-sintered relaxor ferroelectric systems. Fine-grained heterogeneous interfaces microstructures generated through CSP improve polarization reversibility, dielectric breakdown strength, and energy-storage efficiency.

The CSP has enabled significant improvements in dielectric energy-storage performance through its ability to preserve compositionally sensitive structures and maintain dense ceramic architectures at substantially reduced processing temperatures.^34^ Numerous studies have reported enhanced recoverable energy density and improved energy-storage efficiency in CSP-derived relaxor ferroelectrics compared with conventionally processed counterparts.^133,134^ Such improvements have been observed across a broad range of dielectric systems and demonstrate the effectiveness of CSP as a processing platform for advanced capacitor materials.^28,135^ The fundamental differences in polarization behavior and energy-storage mechanisms between conventional ferroelectrics and cold-sintered relaxor systems are illustrated schematically in Fig. 9.

Dielectric breakdown strength remains one of the most important performance metrics because recoverable energy density increases approximately with the square of the applied electric field.^80,133,136^ Consequently, even modest improvements in breakdown strength can produce substantial gains in energy-storage capability. Many CSP-derived ceramics exhibit enhanced breakdown performance, contributing directly to increased recoverable energy density and improved device reliability under high-field operation.^99,137^ The enhanced breakdown strength frequently reported in CSP-derived ceramics is commonly attributed to the combined effects of grain refinement, reduced critical flaw size, improved compositional homogeneity, and suppression of thermally induced defect formation.^135,138^ Because dielectric breakdown is highly sensitive to local electric-field concentrations, the interface-rich microstructures generated during CSP may contribute to a more uniform field distribution and delayed breakdown initiation.^139^

Frequency and temperature stability are equally important for practical deployment. Many cold-sintered relaxor ceramics exhibit relatively stable dielectric responses over broad frequency and temperature ranges, supporting reliable operation under varying service conditions.^140,141^ Stable hysteresis behavior, limited frequency-dependent degradation, and improved operational consistency have been reported in several CSP dielectric systems, further demonstrating their suitability for high-power capacitor applications.^99^ Post-processing treatments frequently play an important role in achieving optimal dielectric performance. Appropriate post-annealing conditions can improve structural stability and enhance polarization behavior while preserving the benefits of low-temperature densification.^66^ Careful optimization of these treatments remains an important aspect of dielectric materials development.

Beyond optimized cold-sintered NaNbO_3_-based relaxors have been reported to achieve recoverable energy densities exceeding several J cm^−3^ with energy efficiencies above 80–90%, whereas conventionally processed counterparts often exhibit lower breakdown strength and reduced polarization reversibility under comparable testing condition,^29^ CSP has been successfully applied to a wide variety of dielectric and ferroelectric materials, including BaTiO_3_-based ceramics,^142^ Bi-based relaxors,^30^ microwave dielectrics,^143,144^ and multilayer dielectric structures.^10,138^ Representative dielectric and energy-storage performances reported for cold-sintered ceramic systems are summarized in Table 4. These studies demonstrate the broad applicability of CSP across diverse dielectric material classes and highlight its potential for next-generation capacitor technologies. The ability to process dielectric materials at low temperatures also creates opportunities for integrated electronic systems. Reduced processing temperatures expand compatibility with thermally sensitive components and facilitate the fabrication of advanced capacitor architectures that are difficult to realize using conventional ceramic processing routes.^145^ Such capabilities are particularly attractive for miniaturized electronics, embedded capacitors, and multifunctional device platforms.

**Table 4:** Representative dielectric and energy-storage ceramic systems processed through CSP together with their microstructural and functional characteristics

Material system	Relative density (%)	Grain size	Dielectric constant (*ε*_r_)	Breakdown strength (kV cm^−1^)	Recoverable energy density (J cm^−3^)	Efficiency (%)	Processing temperature (°C)	Key ref.
NaNbO_3_-based relaxor ceramics	92–98	Submicron	300–1200	250–450	2.0–5.5	75–92	120–300	29
Bi_0_._5_Na_0_._5_TiO_3_ (BNT)-based relaxors	90–97	Fine-grained	500–2500	200–500	1.5–6.0	80–95	150–300	130
BaTiO_3_-based ceramics	90–98	Nano/submicron	1000–5000	150–350	0.5–2.0	70–90	100–300	17
Bi-based relaxor ferroelectrics	90–96	Fine-grained	400–1800	200–400	1.5–4.5	80–95	150–350	41
KNN-based ceramics	90–97	Submicron	300–1500	180–400	1.0–3.5	75–90	150–300	13
Microwave dielectric ceramics	88–97	Fine-grained	20–120	N/A	N/A	N/A	150–300	10
Multilayer dielectric architectures	90–98	Controlled layered microstructure	Application-dependent	200–600	1.0–6.5	80–95	<300	137

**Fig. 10:**
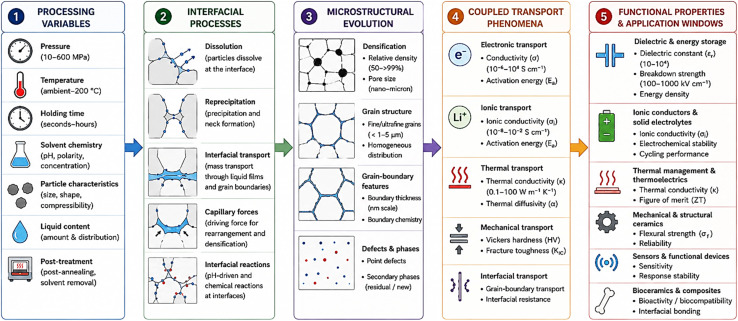
Interface-centered design framework illustrating how processing variables regulate interfacial transport mechanisms, microstructural evolution, and defect chemistry, thereby governing transport behavior and enabling the rational design of multifunctional cold-sintered ceramics for diverse applications.

Despite substantial progress, several challenges remain before widespread technological implementation can be achieved. Long-term reliability under repeated high-field cycling, stability during extended service, and manufacturing reproducibility require further investigation. In addition, a deeper understanding of dielectric breakdown phenomena, polarization dynamics, and degradation mechanisms will be necessary to support predictive materials design and accelerated optimization. The interconnected relationships among processing parameters, microstructural evolution, transport behavior, and functional performance are summarized schematically in Fig. 10. Overall, CSP has emerged as a powerful platform for the development of advanced dielectric and ferroelectric ceramics with competitive energy-storage performance. Enhanced recoverable energy density, improved energy-storage efficiency, high breakdown strength, and excellent operational stability have been demonstrated across numerous material systems. These achievements establish CSP as a promising route for next-generation dielectric energy-storage technologies and provide a foundation for continued advances in high-performance ceramic capacitors. Also, the available evidence indicates that CSP has evolved into a viable processing platform for advanced dielectric ceramics;^35,133^ however, future progress will depend less on demonstrating incremental improvements in energy-storage performance and more on establishing standardized benchmarking against conventionally sintered and thin-film dielectrics under comparable testing conditions. Such comparisons will be essential for identifying the material systems in which CSP offers genuine technological advantages beyond reduced processing temperature.

### 6.2. Thermal transport, thermally functional ceramics, and heat-management applications

Thermal transport plays a critical role in the performance, reliability, and service lifetime of ceramic materials used in electronics, energy systems, aerospace technologies, and high-power devices.^146^ As ceramics increasingly perform multiple electrical, dielectric, thermal, and mechanical functions simultaneously, effective heat management has become a key materials-design objective. Consequently, understanding heat transport in cold-sintered ceramics is essential for both functional optimization and long-term reliability.^25^ In most oxide ceramics, heat conduction is governed primarily by phonon-mediated lattice transport.^23^ Unlike metals, where electrons contribute significantly to thermal conduction, heat transfer in ceramics occurs predominantly through lattice vibrations propagating through the crystal structure. Therefore, thermal conductivity depends strongly on phonon scattering processes occurring throughout the material. Cold-sintered ceramics frequently exhibit thermal transport behavior that differs from conventionally processed materials. The low-temperature nature of CSP generates structural features that modify phonon propagation and alter effective thermal conductivity.^95,147^ As a result, heat transport in these materials is governed not only by intrinsic lattice properties but also by the collective influence of interfaces and structural heterogeneity. A notable characteristic of thermally functional ceramics is that the desired microstructure depends strongly on the target application. While heat-spreading materials require continuous phonon transport and minimal interfacial resistance, thermal barrier and insulating ceramics benefit from enhanced phonon scattering at grain boundaries and interfaces. Consequently, microstructural features that are advantageous for one thermal application may be detrimental to another, highlighting the need for application-specific microstructural design.

A central characteristic of many CSP-derived ceramics is their relatively low thermal conductivity compared with conventionally sintered counterparts.^148–151^ Enhanced phonon scattering increases thermal resistance and shortens effective phonon mean free paths, reducing heat-transfer efficiency. The magnitude of this effect depends on material composition, processing conditions, and the resulting structural architecture.^152,153^ Reduced thermal conductivity can be advantageous in applications where thermal insulation or suppression of heat transport is desired.^154^ A fundamental feature of thermal transport in cold-sintered ceramics is the presence of thermal boundary resistance (Kapitza resistance) at interface-rich grain boundaries. Because CSP generates a high density of chemically and structurally complex interfaces, phonons encounter frequent scattering events that reduce their effective mean free path.^155^ Consequently, thermal conductivity may become increasingly governed by interfacial transport resistance rather than intrinsic lattice conductivity.^156,157^ This transition from bulk-dominated to interface-dominated heat transport represents a defining characteristic of many cold-sintered systems.^158,159^ From a broader perspective, CSP provides opportunities for phonon engineering through deliberate manipulation of grain size, interfacial chemistry, second-phase distribution, and defect populations. Rather than treating reduced thermal conductivity as an unavoidable consequence of low-temperature processing, future research may exploit interface-rich architectures to tailor phonon transport according to application requirements. However, thermoelectric materials, thermal-barrier coatings, and insulating ceramic components may benefit from reduced heat conduction because lower thermal conductivity improves thermal isolation and energy-conversion efficiency. These opportunities have stimulated growing interest in CSP for thermally functional materials. Conversely, many electronic and energy-storage devices require efficient heat dissipation. Components operating under high electrical loading often generate significant amounts of heat during service. If thermal conductivity is insufficient, localized temperature accumulation may occur, leading to thermal stresses, accelerated degradation, and reduced operational reliability. Consequently, optimizing heat removal remains an important consideration in the design of CSP-derived functional ceramics. The literature further indicates that thermal conductivity cannot be interpreted solely as a function of relative density. Residual intergranular phases, interface chemistry, and grain-boundary structure may influence phonon transport as strongly as porosity, particularly in fine-grained CSP-derived ceramics.^118,160,161^ This explains why materials with comparable densities may still exhibit substantially different thermal transport behavior.

Multiphase and heterogeneous ceramic systems introduce additional complexity into thermal transport behavior.^162^ Heat transfer must occur across numerous internal interfaces, and thermal resistance can become strongly dependent on the continuity of conductive pathways throughout the material. As a result, thermal performance is often governed by the collective behavior of the entire architecture rather than by the intrinsic conductivity of individual phases alone.^163,164^ Thermal expansion behavior is also important for thermally functional ceramics.^165^ During heating and cooling cycles, thermal strains develop throughout the material and may influence dimensional stability, mechanical reliability, and interfacial integrity. These effects become particularly important in complex architectures subjected to repeated thermal cycling or harsh service environments. Recent research has increasingly focused on the development of ceramics with application-specific thermal behavior. Rather than simply maximizing or minimizing thermal conductivity, current strategies seek to tailor heat transport according to the requirements of a given application.^14^ This approach reflects the growing recognition that thermal management must be integrated with broader functional objectives, including electrical performance, structural reliability, and device compatibility. Despite substantial progress, several challenges remain. Quantitative relationships between processing conditions and thermal transport properties are not yet fully established for many ceramic systems. In addition, the long-term stability of thermal performance under extended operation and repeated thermal cycling remains insufficiently understood. Addressing these issues will be important for the deployment of CSP-derived ceramics in demanding technological environments. In general, CSP provides a versatile platform for tailoring thermal transport and enabling thermally functional ceramic materials.^57^ By expanding the range of achievable thermal behaviors and supporting application-specific heat-management strategies, CSP offers new opportunities for the development of advanced ceramics for electronics, energy technologies, aerospace systems, and multifunctional devices. Overall, current evidence suggests that CSP provides unique opportunities for engineering thermal transport through interface design rather than density optimization alone.^148,166^ Future studies should therefore place greater emphasis on quantitatively separating the respective contributions of porosity, grain boundaries, and residual interfacial phases to thermal conductivity in different ceramic systems.

### 6.3. Cold-sintered ionic ceramics, solid-state electrolytes, and electrochemical systems

**Fig. 11:**
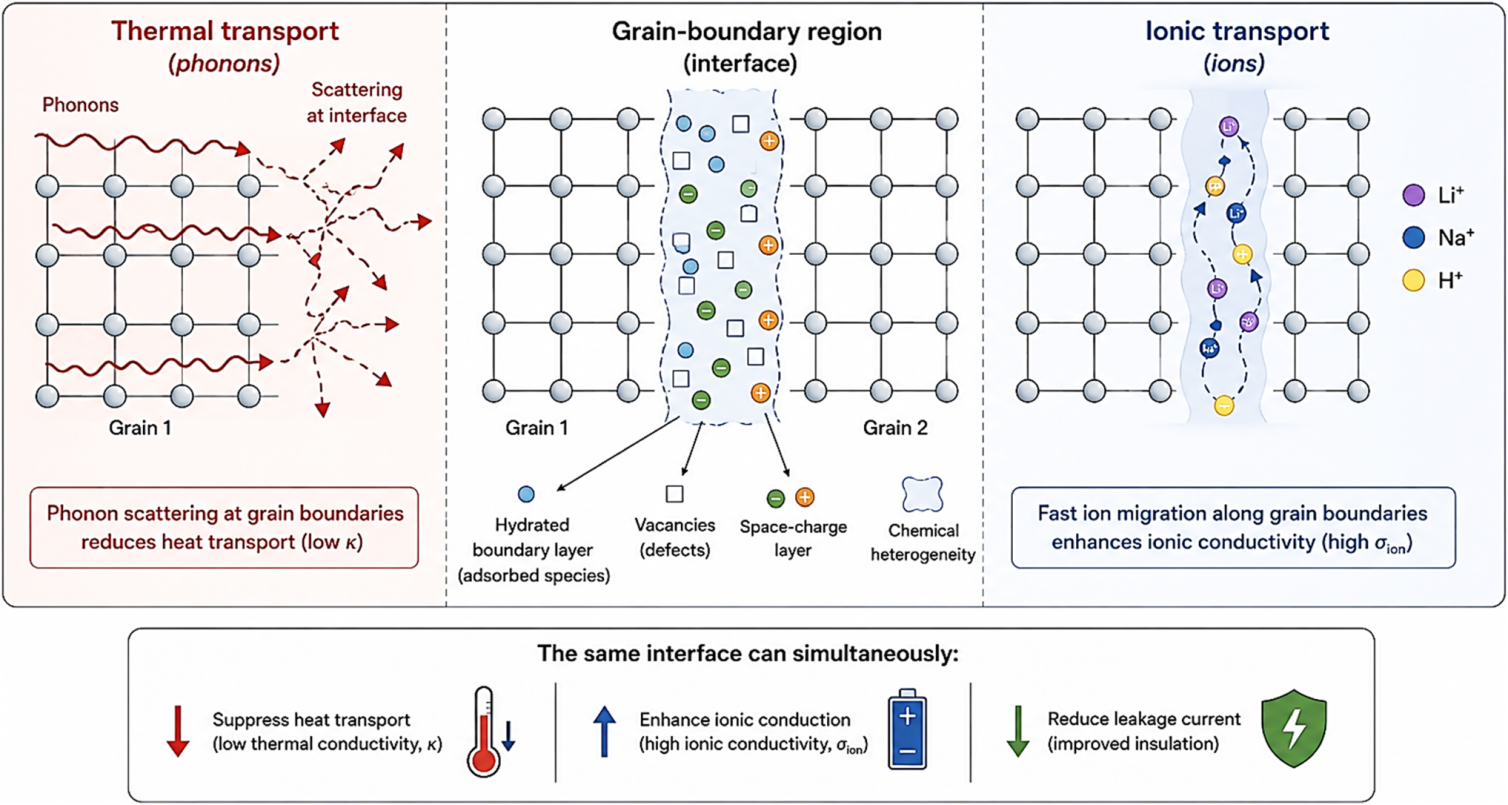
Schematic illustration of coupled thermal and ionic transport phenomena across grain boundaries in cold-sintered ceramics. Interface-rich microstructures may simultaneously suppress phonon transport while facilitating ionic migration depending on local chemistry and defect architecture.

The growing demand for safe, efficient, and high-performance electrochemical energy-storage technologies has stimulated extensive interest in ceramic ionic conductors for applications including solid-state batteries, fuel cells, electrochemical sensors, ionotronic devices, and separation systems.^167^ Compared with liquid electrolytes, ceramic ionic conductors offer improved thermal stability, mechanical robustness, chemical durability, and operational safety. However, conventional fabrication of dense ionic ceramics typically requires high-temperature sintering, which can introduce compositional instability, volatilization, interfacial degradation, and incompatibility with other device components.^168^ The CSP has emerged as an attractive alternative because it enables ceramic densification at substantially reduced temperatures while preserving compositionally sensitive materials.^24^ The coupling between ionic transport and thermal transport pathways across heterogeneous interfaces microstructures is illustrated schematically in Fig. 11. Ionic conduction in ceramics occurs through the migration of mobile species such as Li^+^, Na^+^, H^+^, and O^2−^. The efficiency of this transport process determines the performance of solid electrolytes and many electrochemical devices.^169^ An important trend emerging from recent studies is that improvements in electrochemical performance do not always scale directly with densification.^85,170^ While higher relative density generally reduces pore-related transport limitations, ionic conductivity is often governed more strongly by grain-boundary chemistry, interfacial resistance, and the continuity of ion-conduction pathways. Consequently, comparable densification levels may result in substantially different electrochemical performances across different electrolyte systems. However, ionic conductivity remains one of the most important metrics for evaluating the suitability of ceramic materials for electrochemical applications. For example, Li_6_._25_Al_0_._25_La_3_Zr_2_O_12_ (Al-LLZO), a ceramic material selected for its high ionic conductivity of 1 × 10^−4^ S cm^−1^ at ambient temperature, has been successfully shaped by CSP.^171^ This demonstrates that CSP can achieve ionic conductivities competitive with conventionally processed materials while operating at significantly lower temperatures.

A major advantage of CSP is its ability to reduce thermal degradation during processing. Many ionic conductors contain volatile or thermally sensitive species that may be lost during conventional sintering. Such compositional changes can alter transport behavior and reduce electrochemical performance.^25^ By operating at significantly lower temperatures, CSP helps preserve chemical composition and phase stability, thereby providing a favorable processing route for advanced ionic ceramics.^76^ Lithium-ion conducting ceramics have received particular attention because of their relevance to all-solid-state battery technologies. Garnet-type electrolytes such as Li_7_La_3_Zr_2_O_12_ (LLZO),^172^ NASICON-type conductors,^97^ and perovskite-derived lithium^75^ ceramics represent some of the most widely investigated systems. These materials often require extremely high sintering temperatures to achieve sufficient density through conventional processing. The CSP offers a potential pathway toward densification while reducing thermal exposure and maintaining compositional integrity.^102^ Sodium-ion conducting ceramics have similarly attracted increasing interest because of the rapid development of sodium-based energy-storage technologies.^173^ Representative thermal and ionic transport characteristics of selected cold-sintered ceramic systems are summarized in Table 5. Many sodium-containing materials experience compositional instability during high-temperature processing. The CSP provides opportunities to preserve sodium content while enabling fabrication of dense ceramic electrolytes suitable for electrochemical applications.^174^ Although CSP suppresses volatilization and compositional degradation in solid electrolytes, interfacial resistance frequently remains a major limitation. In many oxide electrolytes, grain-boundary conductivity can be several orders of magnitude lower than bulk conductivity owing to space-charge effects, local compositional deviations, defect segregation, and residual hydrated interphases.^175^ Consequently, optimization of grain-boundary chemistry may become as important as achieving high density in future CSP-derived solid electrolytes. Future progress may increasingly rely on interfacial design strategies that transform grain boundaries from transport barriers into functional ionic pathways. Controlled segregation, engineered defect chemistry, and chemically tailored intergranular phases offer promising routes for enhancing total ionic conductivity without sacrificing the low-temperature advantages of CSP. Although most published studies report improved ionic conductivity after CSP, the magnitude of improvement varies considerably among different electrolyte chemistries.^65,122,176^ Oxide-based and lithium-containing electrolytes, for example, exhibit different sensitivities to residual intergranular phases, post-annealing treatments, and interface composition. These differences indicate that optimizing electrochemical performance requires tailoring processing conditions to the specific transport mechanisms governing each electrolyte family rather than applying a universal processing strategy.

**Table 5:** Thermal and ionic transport behavior in representative cold-sintered ceramics and the dominant role of interfaces in determining functionality

Material system	Dominant charge carrier	Typical conductivity range	Key interfacial features	Advantages of CSP	Main challenges	Representative applications	Key ref.
Li_7_La_3_Zr_2_O_12_ (LLZO)	Li^+^	10^−6^–10^−4^ S cm^−1^	Interface-rich grain boundaries, suppressed Li volatilization	Composition preservation, low thermal budget	Grain-boundary resistance, secondary phases	Solid-state Li batteries	172
LATP/LAGP NASICON electrolytes	Li^+^	10^−5^–10^−3^ S cm^−1^	Controlled intergranular transport	Reduced processing temperature	Interfacial impedance	Li-ion batteries	97
Na_3_Zr_2_Si_2_PO_12_ (NZSP)	Na^+^	10^−5^–10^−3^ S cm^−1^	Heterogeneous transport pathways	Reduced sodium loss	Grain-boundary blocking	Na-ion batteries	102
Other NASICON-type ceramics	Na^+^	10^−6^–10^−3^ S cm^−1^	Controlled Na retention	Improved phase stability	Full densification	Sodium-ion batteries	174
BaZrO_3_-based proton conductors	H^+^	10^−6^–10^−2^ S cm^−1^	Hydration-sensitive grain boundaries	Reduced thermal degradation	Moisture stability	Protonic ceramic fuel cells	94
BaCeO_3_-based proton conductors	H^+^	10^−5^–10^−2^ S cm^−1^	Hydroxyl-containing interfaces	Electrode compatibility	Chemical stability	Hydrogen separation membranes	23
Ceria-based electrolytes	O^2−^	10^−4^–10^−2^ S cm^−1^	Oxygen-vacancy transport	Dopant preservation	Grain-boundary resistance	Intermediate-temperature SOFCs	170
Composite ceramic electrolytes	Mixed ionic conduction	Material dependent	Multiphase transport networks	Multifunctional integration	Long-term interface stability	Hybrid electrochemical devices	186
Ionotronic ceramics	Ion migration and polarization	Material dependent	Polymer–ceramic ionic pathways	Low-temperature integration	Cyclic reliability	Soft electronics, sensors	56

Proton-conducting ceramics represent another important class of materials. These ceramics are central to protonic ceramic fuel cells, hydrogen-separation membranes, and related electrochemical technologies.^177^ Dense microstructures and stable transport pathways are essential for achieving efficient proton conduction.^178^ The low-temperature nature of CSP may provide advantages for processing such materials while minimizing thermally induced degradation.^23^ Beyond batteries and fuel cells, cold-sintered ionic ceramics are increasingly being explored for electrochemical sensors, ionotronic devices, memristive systems, and electrochemically active functional components.^179^ In these applications, controlled ionic migration governs sensing behavior, resistance switching, signal modulation, and electrochemical response. The versatility of CSP therefore extends beyond energy-storage technologies into a broader range of electrochemical devices.^117^ Electrolyte–electrode compatibility remains a major challenge in many solid-state electrochemical systems. Conventional high-temperature processing can promote undesirable reactions, interdiffusion, and degradation at interfaces between dissimilar materials. By substantially reducing processing temperatures, CSP can alleviate many of these challenges and expand the range of compatible material combinations available for device fabrication.^180^

Despite significant progress, several challenges remain before widespread implementation can be achieved. Simultaneously achieving high density, high ionic conductivity, electrochemical stability, and long-term durability remains difficult for many material systems.^181^ Moisture sensitivity, residual processing effects, and performance degradation during extended operation continue to require careful investigation. In addition, quantitative relationships between processing conditions and electrochemical performance remain insufficiently established for many classes of ionic ceramics.^182^ Future research is expected to focus on improving ionic conductivity, enhancing electrochemical stability, broadening materials compatibility, and developing scalable manufacturing routes for practical devices. Continued advances in electrolyte design, interface optimization, and electrochemical engineering are likely to accelerate the integration of CSP-derived ceramics into next-generation batteries, fuel cells, sensors, and multifunctional electrochemical systems. Overall, CSP provides a versatile low-temperature processing route for ionic ceramics and solid-state electrochemical technologies.^183^ By enabling densification of compositionally sensitive materials while preserving electrochemical functionality, CSP offers significant opportunities for overcoming many limitations associated with conventional high-temperature ceramic processing. The growing range of demonstrated electrochemical applications highlights the considerable potential of CSP for future energy-storage and electrochemical-device technologies. As, the available literature demonstrates that CSP offers a promising route for fabricating dense solid electrolytes at substantially reduced temperatures.^184,185^ Nevertheless, future progress should be evaluated not only by further increasing ionic conductivity but also by improving interfacial stability, long-term electrochemical durability, and compatibility with practical electrode materials under realistic operating conditions.

## 7. Composite architectures, hybrid systems, and multifunctional integration in cold-sintered ceramics

**Fig. 12:**
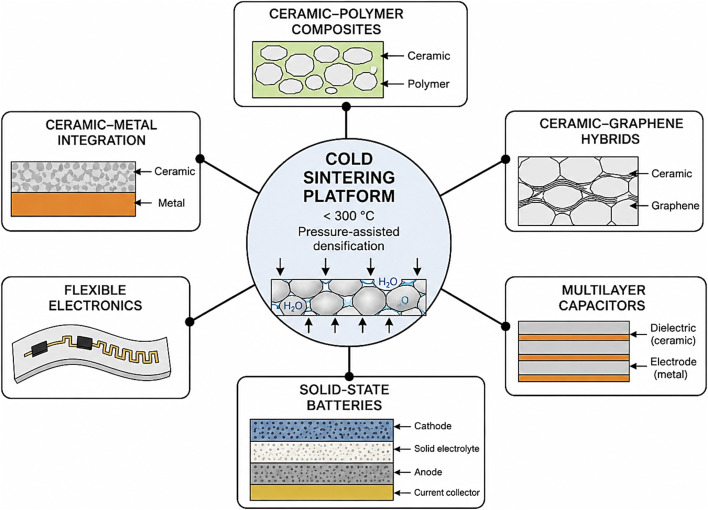
Multifunctional hybrid architectures enabled by low-temperature CSP. The process permits direct integration of ceramics with polymers, metals, carbon-based materials, and electrochemically sensitive phases inaccessible through conventional high-temperature ceramic processing.

A unique advantage of CSP is its ability to integrate materials that are difficult or impossible to combine using conventional high-temperature ceramic processing. Traditional sintering temperatures frequently exceed the thermal stability limits of polymers, low-melting metals, carbon-based materials, and many functional additives.^9^ Consequently, the fabrication of multifunctional architectures incorporating diverse material classes has historically been challenging. By enabling densification at temperatures typically below 300 °C, CSP provides a practical platform for combining ceramics with a wide range of thermally sensitive components within a single structure.^31,40^ Representative multifunctional hybrid architectures enabled by low-temperature CSP are illustrated in Fig. 12. Composite design seeks to combine complementary functionalities that cannot easily be achieved within an individual material.^56^ Through appropriate selection and integration of constituent phases, composite systems can simultaneously provide electrical, thermal, mechanical, dielectric, or electrochemical functionalities.^186^ As a result, composite architectures have become an increasingly important area of CSP research. One of the most actively investigated classes of materials is ceramic–polymer composites.^37^ These systems combine the functional characteristics of ceramic phases with the flexibility, toughness, and process ability of polymers. The low processing temperatures associated with CSP enable direct co-processing of ceramic and polymer components without significant thermal degradation. This capability creates opportunities for flexible electronics, wearable devices, embedded functional components, and mechanically compliant ceramic systems. A key observation emerging from the recent literature is that the principal advantage of CSP in composite systems lies not simply in enabling the co-processing of dissimilar materials, but in preserving their individual functionalities while establishing effective interfacial coupling.^56,103,185^ Consequently, the success of CSP-derived composites should be evaluated by the degree of multifunctional integration achieved rather than by densification alone.

The quality of integration between ceramic and polymer phases is critical for achieving desirable multifunctional performance. The CSP facilitates intimate contact between dissimilar materials through pressure-assisted, solvent-mediated consolidation while preserving the structural integrity of temperature-sensitive polymer components.^135,187^ Consequently, ceramic–polymer composites have emerged as one of the most promising application areas for CSP.^56^ Carbon-containing hybrid systems represent another important category of CSP-enabled materials.^103^ Graphene, carbon nanotubes, graphite, and related carbon-based materials offer unique combinations of electrical, thermal, and mechanical properties.^188,189^ These materials often degrade under conventional ceramic processing conditions but can be incorporated successfully using low-temperature CSP routes. Such hybrid systems have attracted considerable interest for sensing technologies, conductive ceramics, electromagnetic applications, and multifunctional devices.^190^ Metal–ceramic hybrid architectures constitute a further area of development. Conventional ceramic processing frequently limits direct integration with metallic conductors because of oxidation, melting, or interdiffusion at elevated temperatures. The CSP substantially reduces these challenges and enables incorporation of metallic electrodes, conductors, and current collectors within ceramic structures.^7,191^ This compatibility is particularly attractive for multilayer electronics, sensors, and integrated functional devices.

The low-temperature nature of CSP also facilitates the fabrication of multilayer and heterogeneous architectures.^138,192^ Ceramic layers can be combined with conductive, dielectric, polymeric, metallic, or electrochemically active components without exposure to extreme thermal conditions. As a result, CSP is increasingly being explored for advanced packaging technologies, embedded electronics, and multifunctional integrated systems. Representative multifunctional composite and hybrid systems enabled by CSP are summarized in Table 6. Multifunctional integration has become a major motivation for composite design. Modern devices frequently require multiple functionalities to coexist within a single platform. The CSP enables the simultaneous incorporation of diverse material classes while maintaining processing compatibility, thereby supporting the development of highly integrated ceramic systems capable of performing multiple roles simultaneously. However, the literature also reveals important trade-offs associated with multifunctional integration. Strategies that improve electrical or ionic performance may simultaneously reduce mechanical integrity or thermal stability because of differences in thermal expansion, elastic modulus, or interfacial compatibility between constituent phases.^193,194^ These competing requirements indicate that composite design should prioritize overall system performance rather than maximizing a single functional property.

**Table 6:** Representative multifunctional hybrid systems enabled through low-temperature CSP and their targeted applications

Composite architecture	Constituent materials	Interfacial function	Key advantages of CSP	Potential applications	Key ref.
Ceramic–polymer composites	BaTiO_3_–polymer, Al_2_O_3_–polymer	Mechanical compliance and dielectric coupling	Prevents polymer degradation during densification	Flexible electronics, wearable devices	16
Ceramic–carbon hybrids	Ceramic–graphene	Electrical transport enhancement	Preserves carbon nanostructures	Sensors, conductive ceramics	189
Ceramic–CNT composites	Ceramic–carbon nanotubes	Conductive network formation	Avoids CNT oxidation	Electromagnetic shielding, multifunctional electronics	190
Ceramic–graphite systems	Oxide ceramics–graphite	Thermal and electrical pathway engineering	Maintains graphite integrity	Thermal management devices	37
Ceramic–metal hybrids	Ceramics–Ag, Cu, Ni	Electrical conduction and current collection	Reduces oxidation and interdiffusion	Embedded electronics	191
Multilayer ceramic architectures	Dielectric–electrode stacks	Functional layer integration	Co-firing compatibility at low temperature	Capacitors, RF devices	192
Electrochemical hybrid systems	Electrolyte–electrode composites	Coupled ionic/electronic transport	Interface preservation	Batteries, fuel cells	34
Hierarchical composites	Layered and gradient structures	Multiscale transport engineering	Tailored multifunctionality	Smart materials	187
Additively manufactured CSP composites	Printed ceramic–polymer–metal systems	Integrated multifunctionality	Post-print densification below 300 °C	Customized electronic devices	231

Hierarchical composite architectures represent an emerging direction in the field. Rather than relying solely on homogeneous phase mixtures, current strategies increasingly employ layered structures, gradient architectures, aligned fillers, and interconnected networks.^117,195^ These approaches provide additional flexibility for tailoring overall system performance while minimizing compromises between competing design requirements. The compatibility of CSP with additive manufacturing technologies further expands design possibilities.^7^ Conventional ceramic additive manufacturing often requires post-sintering temperatures that are incompatible with embedded functional materials. In contrast, CSP offers opportunities for densifying printed ceramic structures while preserving incorporated polymers, conductive phases, and other temperature-sensitive components.^56^ This capability may enable the fabrication of complex multifunctional devices with highly customized geometries. The next generation of CSP-derived composites is expected to move beyond simple phase mixtures toward architected multifunctional systems. Hierarchical structures incorporating aligned conductive pathways, gradient interfaces, interconnected transport networks, and spatially programmed functionalities may enable simultaneous optimization of electrical, thermal, mechanical, and electrochemical performance. The compatibility of CSP with temperature-sensitive materials also creates opportunities for ceramic metamaterials and architected functional systems whose properties arise from structural organization rather than chemical composition alone.^20^

In spite of substantial progress, several challenges remain. Long-term stability of interfaces between dissimilar phases, mechanical compatibility among constituent materials, manufacturing reproducibility, and large-scale fabrication all require further investigation. Successful multifunctional integration depends not only on the selection of appropriate constituent materials but also on maintaining stable interfaces throughout service life. Future developments are expected to focus on advanced interface engineering, hierarchical architecture design, multifunctional integration strategies, and scalable manufacturing approaches. Continued progress in these areas is likely to expand the range of composite systems accessible through CSP and accelerate the adoption of cold-sintered materials in practical technologies. Overall, CSP provides a powerful platform for the fabrication of composite and hybrid ceramic systems by enabling low-temperature integration of ceramics with polymers, metals, carbon-based materials, and other functional phases. This capability significantly expands the design space available for multifunctional materials and creates new opportunities for advanced electronic, energy, sensing, and structural applications. Also, current research demonstrates that CSP has expanded from a ceramic densification technique into a versatile platform for multifunctional materials integration.^5,20,108,196^ Future advances are likely to arise from rational composite architecture design in which interfacial compatibility, functional synergy, and manufacturing scalability are optimized simultaneously to meet application-specific performance requirements.

## 8. Advanced characterization techniques and computational modeling of cold-sintered ceramics

**Fig. 13:**
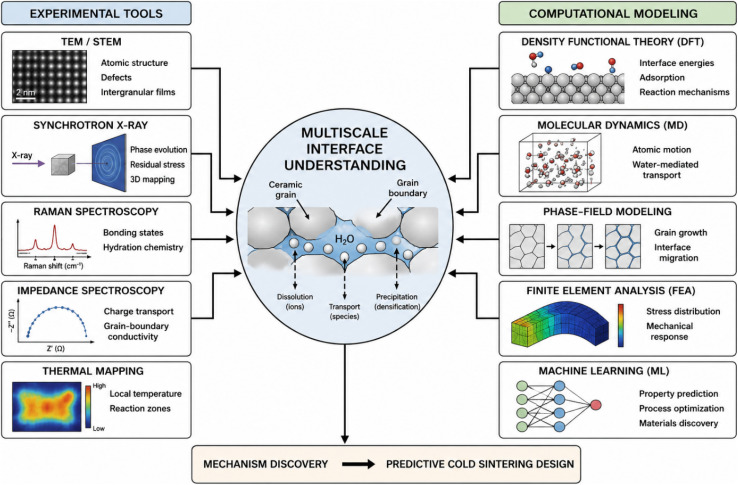
Integrated experimental and computational approaches used for investigating cold-sintered ceramics. Advanced characterization and modeling techniques provide multiscale insights into interfacial structure, transport mechanisms, and microstructural evolution.

The rapid development of CSP has created a need for advanced characterization and modeling approaches capable of resolving complex phenomena that occur during low-temperature densification.^31,90,117^ Unlike conventional ceramic processing, CSP involves the simultaneous action of pressure, transient liquid phases, dissolution–precipitation reactions, and nonequilibrium microstructural evolution.^32^ Consequently, understanding these processes requires analytical tools capable of probing structures and mechanisms across multiple length scales. Many critical features generated during CSP exist at nanometer or atomic dimensions and therefore cannot be fully resolved using conventional bulk characterization techniques. Establishing quantitative relationships among processing conditions, microstructural evolution, and functional properties requires a combination of advanced experimental methods and predictive computational models. A schematic overview of the characterization and modeling framework for cold-sintered ceramics is presented in Fig. 13.

Electron microscopy remains one of the most important techniques for investigating cold-sintered materials. Scanning electron microscopy (SEM) is widely used to evaluate grain morphology, densification behavior, porosity, and fracture characteristics.^49,197,198^ Transmission electron microscopy (TEM) provides direct observation of nanoscale interfaces, amorphous regions, lattice distortions, and secondary phases.^62^ These methods have played a central role in revealing the distinctive interfacial structures produced during CSP. The combination of scanning transmission electron microscopy (STEM) with energy-dispersive X-ray spectroscopy (EDS) or electron energy-loss spectroscopy (EELS) enables high-resolution chemical mapping of interfacial regions.^19^ These techniques provide valuable information regarding local compositional variations, elemental segregation, and nanoscale chemical heterogeneity. Atom probe tomography (APT) is emerging as a powerful complementary technique because it provides three-dimensional atomic-scale compositional information.^199^ Although its application to cold-sintered ceramics remains relatively limited, the method offers significant potential for investigating local chemical distributions and interface chemistry.

Diffraction-based techniques continue to play a fundamental role in structural characterization. Conventional X-ray diffraction (XRD) is routinely employed for phase identification and crystallographic analysis, while synchrotron-based diffraction methods provide enhanced sensitivity to subtle structural features, residual stresses, and minor secondary phases.^49,188,200^ Pair distribution function (PDF) analysis has become increasingly important for investigating short-range structural order and local atomic arrangements that are not accessible through conventional diffraction techniques.^201^ Neutron scattering methods provide complementary structural information and are particularly useful for investigating materials containing hydrogen-related species.^202,203^ Because transient liquid phases frequently participate in CSP processes, neutron-based techniques offer unique opportunities for examining hydration-related structural features. Spectroscopic methods are widely employed to characterize local bonding environments and chemical states.^90,204^ Raman spectroscopy is commonly used to investigate lattice dynamics, structural distortions, and phase stability.^205^ Fourier-transform infrared spectroscopy (FTIR) provides information regarding hydroxyl species and residual processing-related compounds.^18^ X-ray photoelectron spectroscopy (XPS) is frequently used to evaluate oxidation states and surface chemical environments.^206^ Despite significant advances in characterization techniques, most studies still rely on post-mortem analysis after densification has been completed. As a result, many transient processes governing dissolution, interfacial transport, and grain-boundary evolution remain inferred rather than directly observed. Expanding the use of *operando* and *in situ* characterization techniques will therefore be essential for establishing definitive mechanistic evidence and resolving competing interpretations of CSP densification mechanisms.^90,207^

Electrical characterization techniques are indispensable for evaluating transport behavior.^208^ Impedance spectroscopy remains one of the most widely used methods because it enables separation of grain, grain-boundary, and electrode contributions to electrical response.^209^ Equivalent-circuit analysis is commonly employed to quantify the corresponding transport parameters.^90^ Scanning probe techniques provide access to localized functional behavior at nanometer length scales. Methods such as piezoresponse force microscopy (PFM) enable mapping of polarization behavior, electromechanical activity, and local switching characteristics.^210,211^ These approaches are particularly useful for investigating spatially heterogeneous functional responses. Thermal characterization is receiving increasing attention as CSP expands into thermally functional materials.^150^ While laser-flash analysis remains the standard technique for measuring bulk thermal conductivity,^212^ advanced methods such as time-domain thermoreflectance (TDTR)^213,214^ and scanning thermal microscopy^215^ provide spatially resolved information regarding local thermal behavior.

Among the most significant recent developments are *in situ* characterization approaches.^216^ Techniques such as *in situ* synchrotron diffraction, environmental electron microscopy, Raman spectroscopy, and impedance spectroscopy enable direct observation of structural and chemical evolution during densification and post-processing.^78,217–219^ These methods are increasingly contributing to mechanistic understanding of CSP processes. Mechanical characterization methods provide additional insight into local material response and structural integrity. Nanoindentation, microcompression testing, and fracture characterization are widely employed to evaluate mechanical properties at multiple length scales.^89,101^ Computational modeling has become an essential complement to experimental characterization.^220^ Molecular dynamics (MD) simulations are widely used to investigate atomic-scale interactions, interface evolution, and solvent-assisted transport processes.^221^ Density functional theory (DFT) calculations provide information regarding defect energetics, interfacial chemistry, and atomistic stability.^222^ At larger length scales, phase-field modeling has emerged as a powerful framework for simulating microstructural evolution during densification.^223^ These approaches can describe dissolution–precipitation behavior, pore evolution, grain-growth phenomena, and stress development. Finite-element analysis (FEA) is frequently employed to evaluate electric-field distributions, thermal behavior, stress localization, and multiphysics interactions in complex ceramic architectures.^224^ Data-driven methodologies are also attracting increasing attention.^7^ Machine learning and artificial intelligence approaches provide opportunities to identify relationships among processing parameters, microstructural characteristics, and material properties. Such methods may significantly accelerate optimization and materials discovery by reducing experimental trial-and-error efforts.^225^

A particularly promising direction involves *operando* characterization, in which structural, chemical, and transport evolution are monitored during actual device operation rather than under static conditions.^90^*Operando* synchrotron diffraction, impedance spectroscopy, Raman mapping, and thermal imaging may provide unprecedented insight into the dynamic evolution of interfaces and transport pathways in CSP-derived functional ceramics.^226^ Beyond conventional machine learning, emerging physics-informed artificial-intelligence frameworks may provide a powerful route for integrating experimental observations with mechanistic understanding. Such approaches offer the possibility of constructing predictive process–structure–property relationships while maintaining physical interpretability.

Despite substantial progress, important challenges remain. Many CSP phenomena occur across multiple length and time scales, making direct experimental observation difficult. Establishing quantitative links among atomic-scale features, microstructural evolution, and macroscopic performance remains a major objective of ongoing research. Future developments are expected to involve tighter integration of advanced *in situ* characterization, multiscale computational modeling, and data-driven design methodologies. Such approaches may ultimately enable predictive process–structure–property relationships and accelerate the development of application-specific cold-sintered ceramic systems. Representative advanced characterization techniques commonly employed for investigating cold-sintered ceramics are summarized in Table 7. In general, advanced characterization and computational modeling have become indispensable components of modern CSP research. Together, these approaches provide the scientific foundation necessary for understanding densification mechanisms, interfacial evolution, and microstructural development, thereby supporting the transition of CSP toward predictive ceramic engineering and practical technological implementation. Overall, future progress in CSP research will depend not only on developing more sophisticated characterization techniques but also on integrating experimental observations with computational modeling across multiple length and time scales.^220,223^ Such combined approaches will enable quantitative validation of transport mechanisms, improve predictive capabilities, and accelerate the transition from empirical process optimization to mechanism-driven materials design.

**Table 7:** Advanced experimental techniques widely used for investigating structure, transport phenomena, and interface evolution in cold-sintered ceramics

Technique	Information obtained	Length scale	Main advantage	Typical CSP application	Key ref.
SEM	Morphology, porosity, grain growth	Micro-scale	Rapid microstructural analysis	Densification assessment	245 and 246
TEM	Grain-boundary structure	Atomic/nanoscale	Direct interface observation	Interface characterization	247 and 248
STEM-EDS	Elemental mapping	Nanoscale	Segregation analysis	Grain-boundary chemistry	117 and 249
Electron Energy Loss Spectroscopy (EELS)	Local bonding and valence states	Atomic scale	Electronic structure analysis	Defect chemistry	250
Atom Probe Tomography (APT)	3D atomic composition	Atomic scale	Near-atomic chemical mapping	Interface segregation	86
Raman spectroscopy	Bonding and phonon behavior	Microscale	Disorder detection	Phase stability	251
FTIR	Hydroxyl and solvent species	Bulk/local	Detection of residual species	Liquid-phase analysis	252
XPS	Surface chemistry and oxidation states	Nanometer scale	Chemical-state determination	Surface/interface chemistry	253 and 254
Synchrotron XRD	Structural evolution	Atomic/bulk	High-resolution diffraction	*In situ* CSP studies	255 and 256
Pair Distribution Function (PDF)	Local atomic ordering	Atomic scale	Short-range structure analysis	Amorphous interfaces	257
Neutron scattering	Hydrogen-related structures	Atomic/bulk	High penetration depth	Hydrated phases	258 and 259
Impedance spectroscopy	Ionic/electrical transport	Bulk/interface	Grain-boundary separation	Conductivity analysis	90 and 260
Piezoresponse Force Microscopy (PFM)	Local ferroelectric response	Nanoscale	Domain visualization	Polarization mapping	261 and 262
Nanoindentation	Local mechanical behavior	Microscale	Interface mechanics	Grain-boundary strength	89
Time Domain Thermoreflectance (TDTR)^a^	Thermal boundary resistance	Nanoscale	Interface thermal transport	Phonon studies	263
*In situ* Raman/XRD	Dynamic evolution during CSP	Multi-scale	Real-time process monitoring	Mechanism studies	78
Molecular Dynamics (MD)	Atomic transport processes	Atomic scale	Interface dynamics	Solvent-assisted transport	247 and 264
Density Functional Theory (DFT)	Defect energetics	Atomic scale	Fundamental mechanism analysis	Interface chemistry	251 and 265
Phase-field modeling	Microstructure evolution	Mesoscale	Densification simulation	Grain-growth prediction	223
Finite Element Analysis (FEA)	Stress, electric and thermal fields	Macro-scale	Multiphysics modeling	Device optimization	266 and 267
Machine Learning (ML)	Process–property correlations	Multi-scale	Accelerated discovery	CSP optimization	268

a Potential future characterization tool for cold-sintered materials.

## 9. Scalability, manufacturing challenges, sustainability, and future perspectives

**Fig. 14:**
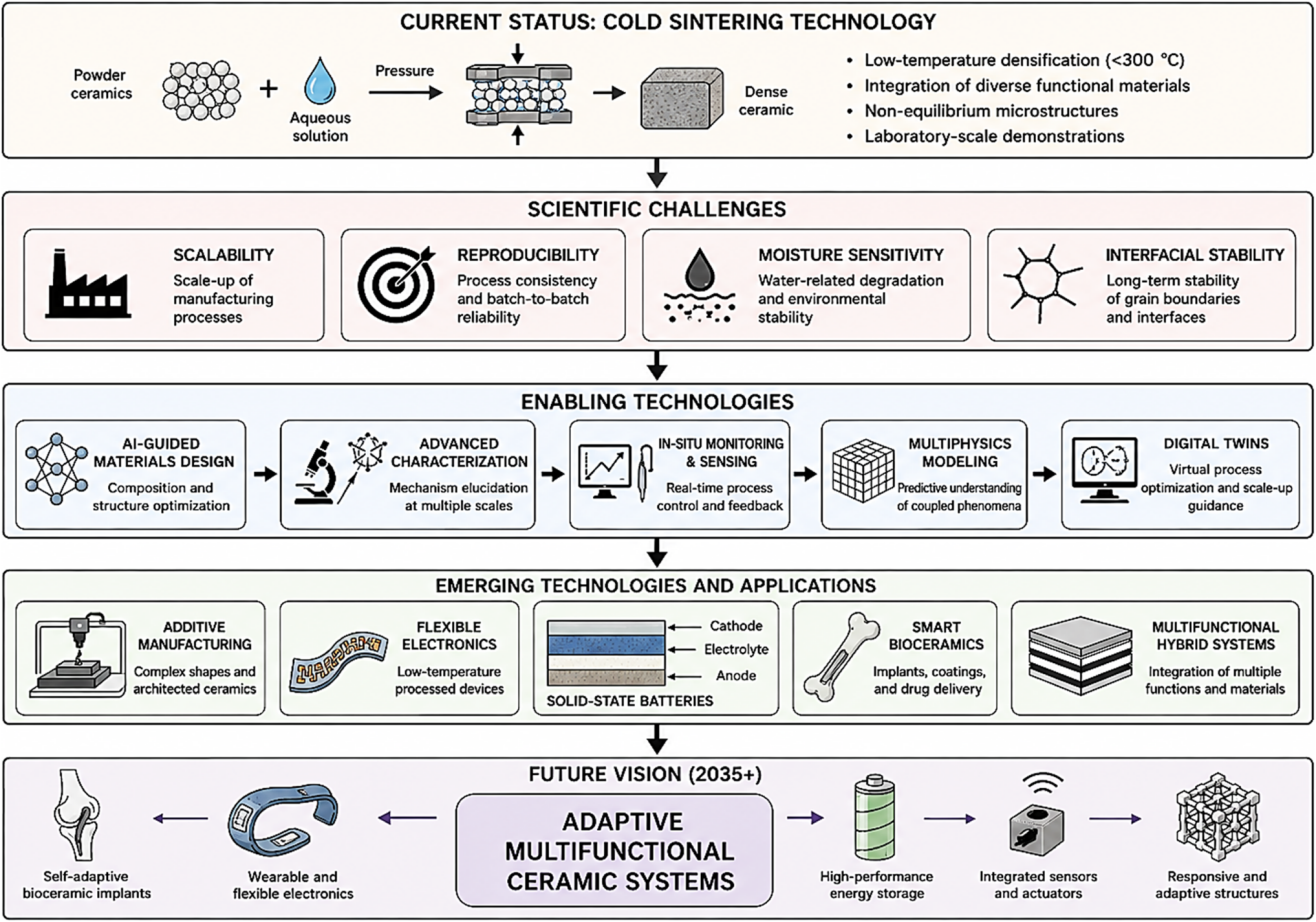
Scientific challenges and future technological directions for cold-sintered ceramics. Future progress will depend on scalable manufacturing, advanced interfacial engineering, multifunctional integration, and predictive materials design strategies.

Despite the rapid growth of CSP research, the transition from laboratory-scale demonstrations to industrial implementation remains one of the most important challenges facing the field.^38,61^ While numerous studies have successfully demonstrated low-temperature densification and attractive functional performance, practical deployment will require scalable manufacturing routes, reliable quality control, long-term stability, and economically viable production strategies.^9,38,40^ A schematic overview of the key scientific challenges and future technological directions for cold-sintered ceramics is presented in Fig. 14. Consequently, future progress will depend not only on scientific advances but also on the development of robust industrial manufacturing frameworks. A major barrier to commercialization is process scale-up.^61^ Most current studies are performed using small laboratory-scale pressing systems,^7,31^ whereas industrial production requires uniform densification across larger components, higher production volumes, and more complex geometries. Maintaining consistent processing conditions during large-scale manufacturing remains challenging and requires improved equipment design, process monitoring, and manufacturing control. A consistent observation across the published literature is that most CSP studies remain confined to laboratory-scale demonstrations involving relatively small and geometrically simple specimens. Although these investigations have successfully established proof-of-concept processing routes, comparatively few studies have systematically evaluated process reproducibility, dimensional scalability, or manufacturing robustness under conditions representative of industrial production.^7,61^

Solvent management represents another important consideration.^227^ Because CSP relies on transient liquid phases during densification, scalable approaches for solvent delivery, distribution, recovery, and removal must be developed.^48^ Effective solvent management will be particularly important for large components and continuous manufacturing processes. Manufacturing reproducibility is equally critical. CSP performance is influenced by numerous processing variables, including powder characteristics, solvent composition, pressure, temperature, and post-processing conditions.^40^ Future industrial implementation will therefore require robust process-control strategies, standardized raw materials, and reproducible manufacturing protocols capable of minimizing batch-to-batch variability. Powder engineering is expected to become an increasingly important aspect of commercial production. Consistent precursor quality, controlled particle characteristics, and reliable feedstock preparation will be essential for ensuring predictable manufacturing outcomes and product performance. For many material systems, post-processing treatments remain necessary to achieve optimal performance.^228,229^ Future manufacturing strategies must therefore balance the benefits of post-treatment with the overall objectives of low-temperature, energy-efficient processing. Minimizing additional processing steps while maintaining performance will be an important consideration for industrial adoption.

Long-term reliability remains a critical requirement for commercial deployment.^19^ Functional ceramics intended for electronic, electrochemical, thermal-management, and structural applications must maintain stable performance under prolonged service conditions. Comprehensive studies addressing aging, environmental stability, cyclic loading, thermal exposure, and operational durability will therefore be essential. Mechanical reliability also requires further investigation, particularly for multilayer devices, hybrid systems, and complex architectures intended for demanding environments.^230^ Establishing standardized reliability-testing methodologies will be important for qualification and certification of future CSP-based products. Standardization represents another major requirement for field maturation. At present, direct comparison among published studies is often difficult because of variations in processing procedures, characterization methods, and performance metrics. The development of widely accepted standards for processing, testing, reporting, and reliability assessment would significantly improve reproducibility and accelerate industrial acceptance. From a sustainability perspective, CSP offers several important advantages compared with conventional ceramic manufacturing. The substantial reduction in processing temperature can significantly decrease energy consumption, shorten production cycles, and reduce associated carbon emissions.^8^ These benefits align well with growing industrial interest in environmentally responsible manufacturing technologies and net-zero production strategies. However, sustainability assessments should extend beyond energy consumption alone. Future evaluations should also consider solvent usage, waste generation, resource efficiency, recyclability, and life-cycle environmental impacts. The development of environmentally benign solvents and closed-loop manufacturing systems may further enhance the sustainability profile of CSP technologies. Similarly, the sustainability advantages of CSP should be evaluated using standardized life-cycle and techno-economic assessment methodologies rather than energy consumption alone. While reduced processing temperatures clearly lower thermal budgets, the overall environmental and economic benefits may also depend on factors such as processing time, applied pressure, solvent recovery, equipment utilization, and production throughput.^43^ Quantitative comparisons using harmonized assessment protocols will therefore be essential for objectively benchmarking CSP against conventional manufacturing routes.

Looking ahead, integration with advanced manufacturing technologies is expected to become increasingly important. Major scientific and technological challenges together with potential future solutions are summarized in Table 8. Additive manufacturing, automated processing platforms, digital manufacturing systems, and smart production environments offer opportunities to expand the design flexibility and scalability of CSP. The ability to densify complex geometries while preserving embedded functional components may enable entirely new classes of ceramic products.^231^ Data-driven optimization and artificial intelligence are also expected to play transformative roles in future development. Machine-learning approaches can accelerate process optimization, identify critical manufacturing parameters, reduce experimental trial-and-error efforts, and support rapid materials discovery.^232^ As datasets continue to expand, AI-assisted design may become an important component of industrial CSP development. Digital twins and predictive manufacturing frameworks represent another promising direction.^233^ By integrating experimental data, computational models, and real-time process monitoring, digital manufacturing platforms may enable predictive control of densification, quality assurance, and process optimization at industrial scales. Digital-twin frameworks may ultimately provide real-time virtual representations of CSP manufacturing processes. By continuously integrating process data, microstructural evolution models, and performance predictions, digital twins could enable adaptive process control, defect prediction, and autonomous optimization during industrial production. Looking further ahead, autonomous research platforms integrating robotics, high-throughput experimentation, machine learning, and computational modeling may substantially accelerate CSP development. Such systems could explore vast multidimensional processing spaces far more efficiently than conventional trial-and-error experimentation. Ultimately, the future success of CSP will depend on establishing reliable process–manufacturing–performance relationships that connect laboratory-scale discoveries with industrial production requirements. Through continued advances in scale-up, reliability assessment, sustainability analysis, process automation, and digital manufacturing, CSP has the potential to evolve from an emerging research technique into a commercially viable manufacturing platform for advanced ceramic technologies. Also, the future success of CSP will ultimately depend not only on continued improvements in material performance but also on the development of robust manufacturing protocols, standardized testing procedures, and economically viable production strategies. Addressing these challenges will be essential for translating laboratory-scale advances into commercially competitive ceramic technologies.

**Table 8:** Major scientific and technological challenges limiting large-scale implementation of cold-sintered ceramics together with potential future solutions^3,4,7,40,61^

Challenge category	Current limitation	Scientific impact	Potential solution	Future research direction	Key ref.
Densification mechanisms	Incomplete understanding of dominant transport pathways	Limits predictive process design	*In situ* characterization and multiscale modeling	Mechanism-based CSP design	269
Grain-boundary engineering	Complex nonequilibrium interfaces	Property variability	Atomic-scale interface characterization	Interface-by-design strategies	87
Ionic conductivity	High grain-boundary resistance	Reduced electrochemical performance	Interface chemistry optimization	High-conductivity electrolytes	185
Mechanical reliability	Weak interfacial cohesion	Reduced durability	Grain-boundary strengthening approaches	Long-life structural ceramics	270
Scale-up manufacturing	Laboratory-scale processing	Limited industrial adoption	Continuous and automated CSP systems	Industrial-scale production	61
Process reproducibility	Sensitivity to processing variables	Batch-to-batch variability	Standardized processing protocols	Manufacturing qualification	271
Solvent management	Liquid distribution and removal challenges	Process inconsistency	Closed-loop solvent systems	Sustainable manufacturing	196
Long-term stability	Aging and environmental degradation	Reliability concerns	Accelerated lifetime testing	Service-life prediction	272
Data availability	Fragmented datasets	Slow optimization	Open CSP databases	Data-driven materials discovery	95
AI integration	Limited predictive capability	Slow materials development	Machine-learning frameworks	Autonomous process optimization	268
Sustainability assessment	Lack of quantitative metrics	Uncertain environmental impact	Life-cycle assessment (LCA)	Net-zero ceramic manufacturing	172
Digital manufacturing	Limited real-time control	Reduced scalability	Digital twins and smart sensing	Industry 4.0 CSP platforms	31

## 10. Conclusions and outlook

Cold sintering has emerged as one of the most significant advances in ceramic processing over the past decade, demonstrating that dense ceramic materials can be fabricated at temperatures far below those required by conventional sintering routes. By combining pressure-assisted consolidation with transient liquid-phase transport, CSP provides a fundamentally different pathway for densification while expanding the range of materials and architectures accessible to ceramic manufacturing. Several key conclusions can be drawn from the current state of the field. First, CSP is no longer viewed solely as a low-temperature densification technique. Instead, it has evolved into a broader materials-engineering platform capable of controlling interfaces, microstructures, phase assemblages, and functional performance. This capability has enabled the development of advanced dielectric materials, electrochemical ceramics, thermally functional systems, and multifunctional hybrid architectures that are difficult to achieve using traditional processing methods. Second, the unique compatibility of CSP with temperature-sensitive materials has significantly expanded opportunities for multifunctional integration. The ability to combine ceramics with polymers, metals, carbon-based materials, and other functional phases opens new possibilities for next-generation electronic, energy-storage, sensing, and integrated-device technologies. Third, the field has increasingly shifted from demonstrating feasibility toward understanding mechanisms and establishing predictive design principles. Advances in characterization, modeling, and data-driven analysis are providing deeper insight into densification behavior, microstructural evolution, and performance optimization. These developments are laying the foundation for more rational and application-oriented materials design.

Despite these achievements, several challenges continue to limit widespread implementation. Fundamental questions remain regarding long-term stability, processing reproducibility, reliability under service conditions, and large-scale manufacturing. Addressing these challenges will be essential for translating laboratory-scale successes into commercially viable technologies. Looking ahead, future progress is expected to be driven by the convergence of advanced manufacturing, artificial intelligence, multiscale modeling, and sustainable processing strategies. Greater integration of experimental data, predictive simulations, and digital manufacturing tools may enable accelerated materials discovery and improved process optimization. At the same time, increasing attention to scalability, standardization, and environmental sustainability will be necessary for industrial adoption. More broadly, CSP represents a conceptual shift in ceramic manufacturing. Rather than relying primarily on temperature as the dominant processing variable, CSP enables functionality to be engineered through interfaces, local chemistry, and transport pathways. In this sense, the future of CSP may extend beyond low-temperature densification toward a new paradigm of interface-programmed ceramic materials, where thermal, electrical, ionic, and mechanical responses are deliberately designed through interfacial architectures. The convergence of advanced characterization, multiscale modeling, artificial intelligence, and scalable manufacturing may ultimately transform CSP from an emerging processing technique into a foundational platform for next-generation ceramic engineering.

**Fig. 15:**
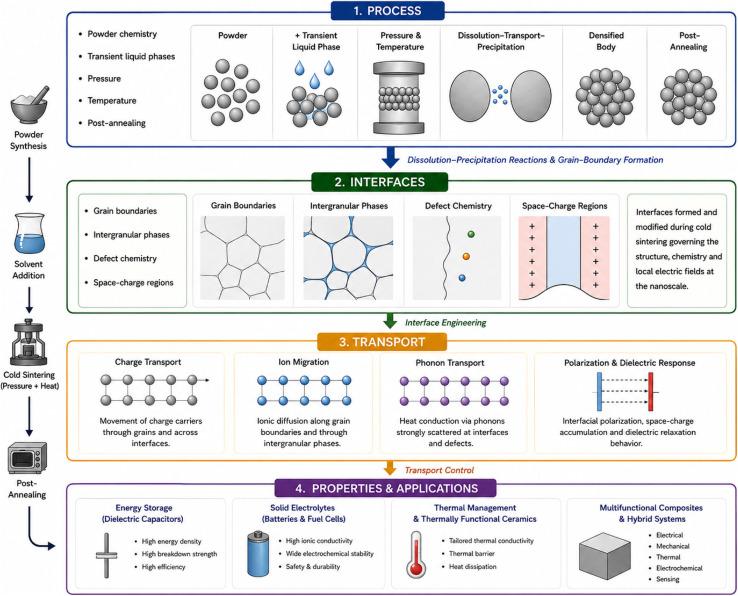
Process–interface–transport–property paradigm in cold-sintered ceramics. The figure illustrates how powder chemistry and transient liquid phases govern dissolution–precipitation reactions and grain-boundary formation, which subsequently control interfacial transport processes and multifunctional properties in cold-sintered ceramic systems.

A unifying perspective emerging from this review is that the functional behavior of cold-sintered ceramics can be understood through a process–interface–transport–property paradigm. In this framework, powder chemistry, transient liquid-phase composition, and processing conditions govern dissolution–precipitation reactions and grain-boundary formation during densification. These engineered interfaces subsequently control ionic migration, dielectric polarization, electrical transport, phonon scattering, and electrochemical behavior, ultimately determining multifunctional performance. Fig. 15 summarizes this concept and highlights the transition of CSP from a low-temperature densification technology toward an interface-programmed ceramic engineering platform. Future advances are expected to increasingly focus on deliberate interfacial design as a route for tailoring transport pathways and achieving application-specific functionalities. Overall, the literature reviewed in this work indicates that CSP has evolved from a low-temperature densification technique into an interface-engineering platform capable of tailoring microstructure, transport behavior, and multifunctional performance across diverse ceramic systems. At the same time, the evidence demonstrates that no single processing strategy or mechanistic model is universally applicable. Future progress will therefore depend on developing quantitative, experimentally validated frameworks that link processing conditions to interfacial evolution and ultimately to functional performance. Such advances will be essential for translating CSP from laboratory-scale demonstrations to reliable industrial manufacturing.

## Author contributions

Kaveh Rahimi Mamaghani: conceptualization, investigation, data curation, validation, formal analysis, writing – original draft, and visualization. Nader Parvin: resources, writing – review & editing, supervision, project administration, and funding acquisition.

## Conflicts of interest

The authors declare that they have no known competing financial interests or personal relationships that could have appeared to influence the work reported in this paper.

## Data Availability

This article is a review of previously published literature. No new experimental data, computational data, or software were generated as part of this work. All information supporting the findings of this study is available within the article and the cited references.
